# Searching for Old and New Small-Molecule Protein Kinase Inhibitors as Effective Treatments in Pulmonary Hypertension—A Systematic Review

**DOI:** 10.3390/ijms252312858

**Published:** 2024-11-29

**Authors:** Magdalena Jasińska-Stroschein, Paulina Glajzner

**Affiliations:** Department of Biopharmacy, Medical University of Lodz, ul. Muszyńskiego 1, 90-151 Lodz, Poland; paulina.glajzner@umed.lodz.pl

**Keywords:** animal modeling, clinical trials, pulmonary arterial hypertension, safety, small-molecule protein kinase

## Abstract

Treatment options for pulmonary arterial hypertension (PAH) have improved substantially in the last 30 years, but there is still a need for novel molecules that can regulate the excessive accumulation of pulmonary artery smooth muscle cells (PASMCs) and consequent vascular remodeling. One set of possible candidates are protein kinases. The study provides an overview of existing preclinical and clinical data regarding small-molecule protein kinase inhibitors in PAH. Online databases were searched from 2001 to 2023 according to PRISMA. The corpus included preclinical studies demonstrating alterations in at least one PH-related parameter following chronic exposure to an individual protein kinase inhibitor, as well as prospective clinical reports including healthy adults or those with PAH, with primary outcomes defined as safety or efficacy of an individual small-molecule protein kinase inhibitor. Several models in preclinical protocols (93 papers) have been proposed for studying small-molecule protein kinase inhibitors in PAH. In total, 51 kinase inhibitors were tested. Meta-analysis of preclinical results demonstrated seralutinib, sorafenib, fasudil hydrochloride, and imatinib had the most comprehensive effects on PH with anti-inflammatory, anti-oxidant, and anti-proliferative potential. Fasudil demonstrated more than 70% animal survival with the longest experimental period, while dasatinib, nintedanib, and (R)-crizotinib could deteriorate PAH. The substances targeting the same kinases often varied considerably in their activity, and such heterogeneity may be due to the variety of causes. Recent studies have addressed the molecules that affect multiple networks such as PDG-FRα/β/CSF1R/c-KIT/BMPR2 or FKBP12/mTOR. They also focus on achieving a satisfactory safety profile using innovative inhalation formulations Many small-molecule protein kinase inhibitors are able to control migration, proliferation and survival in PASMCs in preclinical observations. Standardized animal models can successfully reduce inter-study heterogeneity and thereby facilitate successful identification of candidate drugs for further evaluations.

## 1. Introduction

Pulmonary hypertension (PH) is a pathophysiological and life-treating condition with a multifactorial etiology and poor prognosis. The patients can present with a variety of cardiovascular and respiratory symptoms associated with poor exercise capacity, right ventricle (RV) dysfunction, and hypertrophy. The condition can affect the small pulmonary arterioles, where it is classified as pulmonary arterial hypertension (PAH) (clinical group 1) [1,2,3]. Although PAH has a low prevalence, it can result in heart failure and death, and thus requires comprehensive management [1].

The progression of deleterious pulmonary vascular remodeling in PAH is mediated by several dysfunctions of both the pulmonary endothelium and vascular smooth muscle, accompanied by abnormalities in the inflammatory and immune system. Most current treatments aim to remediate three well-recognized and described drivers, including decreased levels of endogenous vasodilators (prostacyclin, phosphodiesterase type 5 inhibitors—PDE-5is, soluble guanylate cyclase stimulators—sGCSs) and elevated levels of endogenous vasoconstrictors (endothelin receptor antagonists—ERAs). Even so, some patients remain refractory to treatment, even three-drug combination therapy based on different mechanisms [4]. Recent reviews show that many drugs currently under evaluation for treating PAH improve pulmonary vascular hemodynamics and RV structure [5]. It has now been recognized that a critical role in disease progression can be played by pulmonary vascular remodeling driven by abnormal vascular cell proliferation and apoptosis [6]. Hence, recent research efforts have been made to identify and develop novel therapeutic approaches that can not only influence vasoconstriction, as with existing therapies, but can manage the excessive accumulation of pulmonary artery smooth muscle cells (PASMCs) and consequent vascular remodeling.

For example, recent phase 2 and 3 clinical trials of sotatercept, a first-in-class fusion protein, indicate that it is possible to substantially improve the condition of patients with PAH by restoring pulmonary vascular homeostasis toward growth-inhibiting and pro-apoptotic signaling [7]. Recently, preclinical studies have also obtained promising results for another protein, seralutinib; these warranted further clinical trials which are currently in progress. Seralutinib is a small-molecule kinase protein inhibitor that targets pathways implicated in vascular remodeling in PAH, including platelet-derived growth factor receptors (PDGFRs) [8]. About ten years ago, another PDGFR blocker, the tyrosine kinase inhibitor (TKI) imatinib, obtained disappointing results in trials and due to safety concerns it was not approved [9]. Therefore, current research focuses on the development of novel therapeutic approaches which target the lung with the aim of reducing the adverse effects of PDGF-linked molecules [10]. Such efforts also addressed imatinib, and this again raises the question of whether targeting different protein kinases can effectively and safely control pulmonary artery remodeling in PAH.

Protein kinases play a significant regulatory role in cell biology. Their activation can initiate a cascade of downstream signaling pathways involved in cellular proliferation, differentiation, and survival. For this reason, protein kinases play a significant role in the pathogenesis of a number of malignancies in addition to various autoimmune, inflammatory, nervous, and cardiovascular diseases. About 25% of drug development efforts may target these enzymes [11], and hundreds of protein kinase inhibitors in preclinical and clinical trials worldwide may be effective against neoplasms as well as non-neoplastic diseases, including pulmonary arterial hypertension [12].

The primary objective of this paper is to provide an overview of the current state of knowledge regarding the activity of a wide spectrum of small-molecule protein kinase inhibitors, with particular emphasis on their potential to manage PAH. The paper analyzes the hemodynamic, echocardiographic, histomorphometric, and biochemical findings from preclinical trials and discusses animal survival and the rationale for further clinical assessment. Secondarily, it also explores the potential errors occurring when translating results from animal experiments to humans. For this reason, this paper also provides a brief summary of clinical studies on the discussed molecules and PAH.

## 2. Results

### 2.1. Article Search

**Figure 1:**
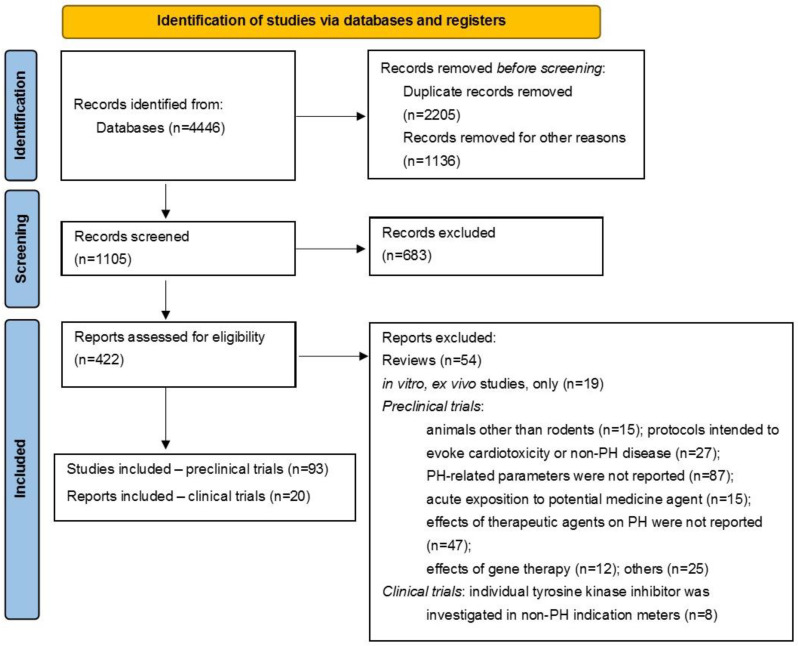
PRISMA 2020 flowchart of the preclinical and clinical study selection process.

An initial search revealed 4446 items. Of these, 442 papers were considered potentially relevant. In total, 1105 records were screened, and finally, 93 preclinical protocols and 20 clinical trials were included in the review. The preclinical trials included a vehicle group (animals with PH, placebo) and a treatment group (animals with PH, therapeutic intervention) and described alterations in at least one PH-related parameter caused by chronic exposure to an individual small-molecule protein kinase inhibitor [13,14,15,16,17,18,19,20,21,22,23,24,25,26,27,28,29,30,31,32,33,34,35,36,37,38,39,40,41,42,43,44,45,46,47,48,49,50,51,52,53,54,55,56,57,58,59,60,61,62,63,64,65,66,67,68,69,70,71,72,73,74,75,76,77,78,79,80,81,82,83,84,85,86,87,88,89,90,91,92,93,94,95,96,97,98,99,100,101,102,103,104,105]. The clinical trials comprised prospective reports of different statuses (i.e., recruiting, ongoing, completed, or terminated) performed on adult subjects (healthy or with PAH); the primary outcomes were safety or efficacy (inter alia 6MWD, hemodynamic parameters) associated with chronic exposure to an individual small-molecule protein kinase inhibitor [9,106,107,108,109,110,111,112,113,114,115,116,117,118,119,120,121,122,123,124,125] (Figure 1).

### 2.2. Quality Assessments of Preclinical Trials

As demonstrated in Figure S1 (Supplementary Materials), most of the preclinical studies included in the current review manifested unclear risk of bias. Most studies included in the meta-analysis did not influence the significance of the leave-one-out sensitivity analysis according to individual PH-related parameters (Table S1). The exceptions were: right ventricle systolic pressure (RVSP) parameter (among activin receptor-like kinase (Alk) or fibroblast growth factor receptor (FGFR) blockers), blood pressure (BP) (vascular endothelial growth factor receptor (VEGFR) blockers) or cardiac output (CO) (BCR-ABL or FGFR blockers). In each of these cases, analyses were repeated after removing one from the total number of studies. As summarized in Table S2, most of the experimental protocols demonstrated an absence of publication bias in relation to particular parameters associated with PH, as indicated by a non-significant Egger (*p* > 0.05) test result and low possibility of missing studies (trim and fill procedure). This was also true when analyzing results in subgroups defined by a particular target for the therapeutic agent (e.g., the kinase family).

### 2.3. Animal Data Review and Synthesis

#### 2.3.1. Characteristics of Included Studies

The review included a total of 2547 animals examined in 93 preclinical studies and 215 interventions, with intervention being defined as an individual comparison between the treatment and placebo groups. Most experimental protocols used rats (N = 170/215; 79.1%) and male subjects (N = 189/215; 87.9%). In these studies, PH was induced by a single intraperitoneal injection of monocrotaline (MCT) at 40–70 mg/kg (49.8%) (clinical group 1, pulmonary arterial hypertension) that may be followed by left pneumonectomy (LP+MCT; 2.3%). Approaches based on chronic exposure to hypoxic conditions (10% oxygen) (clinical group 3, PH associated with lung diseases and/or hypoxia) were also commonly used in preclinical studies on small-molecule kinase inhibitors (19.5%). Some studies used a combination of chronic hypoxia and injection of SU5416, a selective inhibitor of VEGF receptor (13.0%). A few models included aortic banding (3.7%) (clinical group 2, PH associated with left heart disease), bleomycin-induced pulmonary fibrosis and PH (2.8%) (clinical group 3, PH associated with lung diseases and/or hypoxia), or genetic modifications (4.6%). The latter approaches could evoke PH due to impaired signaling of bone morphogenetic protein (BMP) (CH+EC-Bmpr−/+; SM22-tet-BMPR2R899X), serotonin (SU+CH+Tph1−/−), or peroxisome proliferator-activated receptor (Tie2 PPARγ−/−).

In 24 out of 93 papers, more than one animal model was used to test the potential efficacy of protein kinase inhibitors. In total, 51 kinase inhibitors were tested, which were further classified into subgroups defined by the kinase family acting as their primary target, the most common substances being fasudil hydrochloride (ROCK) (18.1%), imatinib mesylate (BCR-ABL) (15.3%), sirolimus (mTOR) (8.4%), or sorafenib (VEGFR) (4.2%). The median duration of drug administration was 14 days (IQR; 14–21). Sixty percent of interventions were based on a late (therapeutic) regimen, where an individual drug candidate was administered several days after induction of PH; other protocols were based on an early (preventive) regimen, where the animals were exposed to the PH inductor and treatment at the same time. More detailed information about the studies is provided in Table S3.

#### 2.3.2. Efficacy of Agents Targeted Selected Protein Kinase Pathways

In most of the 93 preclinical studies, the statistical analysis indicated some protein kinase inhibitors improved right ventricle hemodynamics or reduced RV hypertrophy or pulmonary artery (PA) remodeling. For the purposes of the current analysis, the molecules were classified into subgroups based on their target kinase family: receptor protein-tyrosine kinase (ALK, EGF, FGF, VEGF), non-receptor protein-tyrosine kinases (Janus kinase—Jak1/2), protein-tyrosine kinase Abl (BCR-ABL), and protein-serine/threonine kinases (RhoA kinase—ROCK). Two other subgroups were those targeting mammalian target of rapamycin (mTOR) and transforming growth factor (TGF).

**Figure 2:**
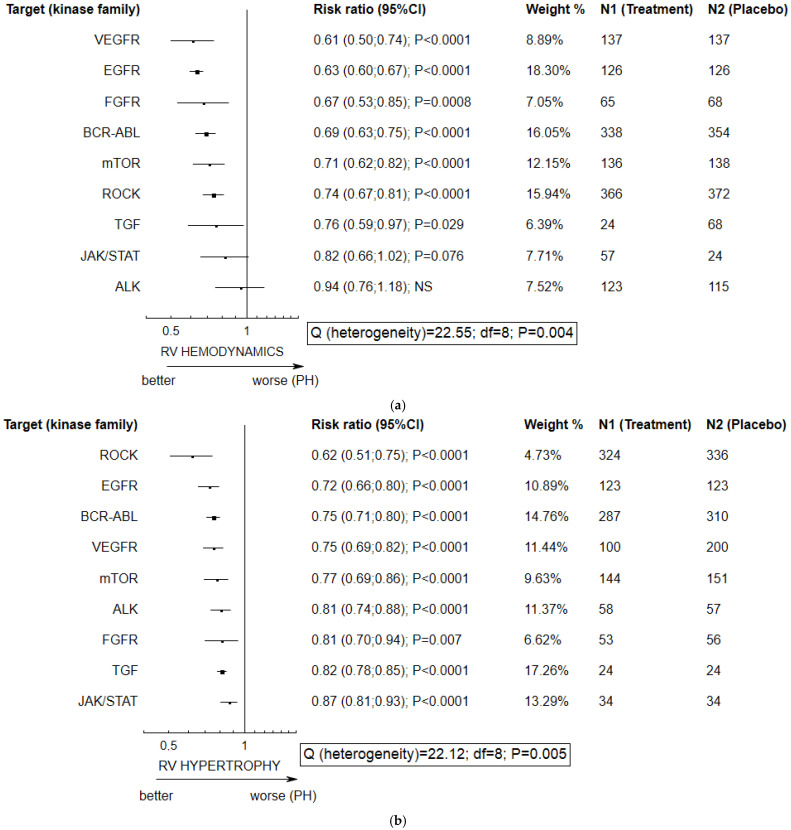

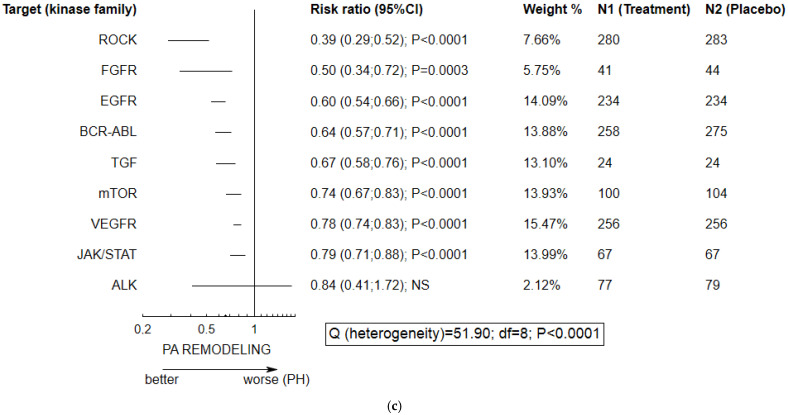
Efficacy of small-molecule protein kinase inhibitors against PH-related features according to primary target (kinase family), as noted in preclinical studies. (**a**) Most therapeutic agents, except for JAK and Alk kinase inhibitors, demonstrated a reversal of RV hemodynamics with a composite end-point, including a decrease in right ventricle systolic pressure (RVSP) and mean pulmonary arterial pressure (mPAP) (**b**) and decrease in RV hypertrophy. (**c**) Pulmonary artery remodeling was non-significantly reversed following exposure to Alk kinase inhibitors only. NS—non-significant. An effect size (R) < 1 (Equation (S2)) indicates a decrease in the mean value of a parameter in PH animals exposed chronically to individual agent as compared to PH subjects treated with placebo; R = 0.50 would indicate an approximately two-fold reversal in PH manifestations. The Q measure (*p* < 0.05) indicates pronounced heterogeneity between subgroups of animals treated with agents targeting different kinases. The analyses were performed according to extracted data addressing individual parameters: the mean (+/−SD, or +/−SEM) and number of animals per group (n). Where the range of animal subjects (e.g., 8–12) was given in an individual study, the lowest number was used; where the study results did not include any data about the number of subjects, the number of subjects at randomization was considered.

The multikinase inhibitor seralutinib (PDGFR; c-Kit; BMPR2), given by inhalation, demonstrated the greatest reduction of right ventricle systolic pressure and/or mean pulmonary arterial pressure as well as RV hypertrophy. Some studies found sorafenib (VEGFR, b-RAF, c-KIT, PDGFR-β) to have a similar effect on PH-linked parameters, but it also significantly increased systemic blood pressure. In addition, fasudil (ROCK) and gefitinib (EGFR) significantly reduced pulmonary artery remodeling. Imatinib (BCR-ABL), sorafenib, seralutinib, and fasudil hydrochloride had the most comprehensive effects on PH, with anti-inflammatory, anti-oxidant, and anti-proliferative potential. Forest plots comparing various kinase inhibitors and placebo with regard to continuous data concerning hemodynamic and hypertrophic parameters (response ratio, RR) are given in Figure 2 and Figure S2 (Supplementary Materials). In Figure 2, the inhibitors are grouped according to their primary target (i.e., BCR-ABL, EGFR, FGFR, VEGFR, TGF, JAK-STAT, mTOR, Alk). The data for individual agents, including multikinase inhibitors, such as seralutinib or BCG-311, are given in the other forest plot in Figure S2. Imatinib, given by inhalation, yielded greater normalization of the hemodynamics and hypertrophy of the right ventricle than administration per os (intragastric). In contrast, dasatinib (BCR-ABL), nintedanib (FGFR), and (R)-crizotinib (Alk) treatment was associated with disease deterioration characterized by more intense PH-related parameters. Also, the selective VEGFR blocker cabozatinib tended to exacerbate pulmonary hypertension (RV hypertrophy), unlike non-selective VEFGR blockers that additionally target PDGFR-β or FGFR 1 (regorafenib), PDGFR-β or c-KIT (sunitinib), or b-RAF, c-KIT, or PDGFR-β (sorafenib) (Table 1 and Table S4, Figure 2 and Figure S2).

**Figure 3:**
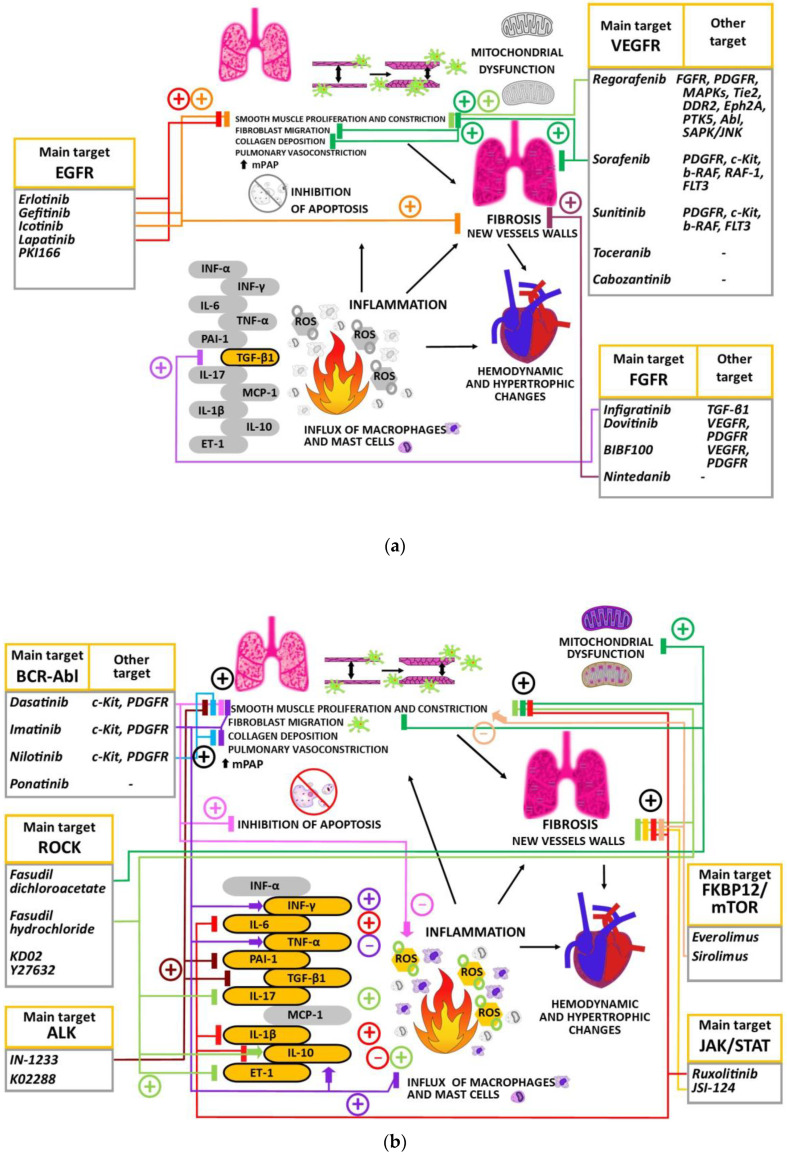

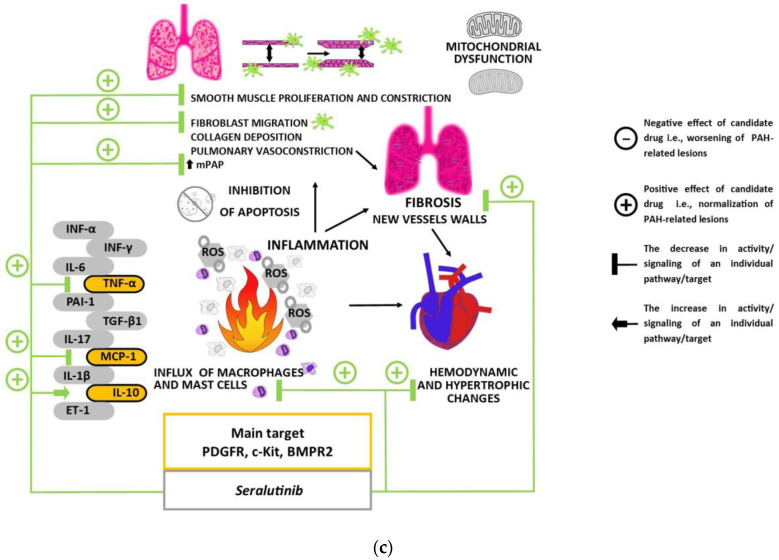
Schematics describing the role of particular small-molecule protein kinases and their inhibitors in PAH microenvironment, including such molecular hallmarks as inflammation, apoptosis, proliferation, or mitochondrial function. Many of these compounds have multidirectional effects. The molecular background involves anti-inflammatory activity by influencing TNF-α, MCP-1, IL-10 factors. (**a**) Molecules that reduce the expression of tyrosine kinase receptors with their main target defined as EGF, FGF, or VEGF receptors, inhibiting the proliferation of smooth muscle cells and, consequently, the process of lung fibrosis. (**b**) Rho A kinase inhibitors normalize PAH-related hemodynamic and hypertrophic lesions in the heart and the process of proliferation in the pulmonary arteries. They can beneficially activate anti-inflammatory cytokines, but decrease vasoconstrictors—endothelin ET-1, pro-inflammatory interleukin IL-17—and reduce oxidative stress. Additionally, fasudil dichloroacetate is responsible for the reduced influx of fibroblasts and inhibition of the process leading to mitochondrial dysfunction. Sirolimus, an inhibitor of the FKBP12/mTOR complex, intensifies the process of proliferation and migration of fibroblasts and yet inhibits the ongoing hypertrophic changes in the heart. Inhibition of pulmonary fibrosis is also a primary mechanism for inhibitors of the JAK/STAT signaling pathway (ruxolitinib). The molecule exerts anti-inflammatory activity (IL-1β, IL-6, IL-10), with slight effect on the PASMC remodeling. The compound IN-1233, an ALK kinase inhibitor, is distinguished by its inhibitory effect on the production of pro-inflammatory factors (TGF-β1, PAI-1). Molecules that block BCR-ABL kinase inhibit PDGF-induced proliferation and migration within smooth muscle cells of vascular walls and reduce collagen deposition in vascular walls. However, imatinib might intensify the inflammatory process (↑INF-γ, TNF-α). Similarly, dasatinib increases the formation of free radicals and does not inhibit hemodynamic and hypertrophic changes in the heart. (**c**) Seralutinib, which targets PDGF/c-KIT/BMP receptor type 2 (BMPR2) signaling, reduces smooth muscle proliferation and fibroblast migration and inhibits the process of narrowing the walls of pulmonary vessels. The molecular background involves anti-inflammatory activity by influencing TNF-α, MCP-1, IL-10 factors. Plus in a circle—positive effect of candidate drugs on the normalization of PAH-related lesions; minus in a circle—negative effect of candidate drugs that manifest the worsening of PAH-related lesions; sharp arrow—increase in activity/signaling of an individual pathway/target; blunt arrow—decrease in activity/signaling of an individual pathway/target. The untargeted pathways/factors by an individual agent are marked in gray [8,19,25,31,74,94,101,105]. ALK—activin receptor-like kinase; BMPR2—bone morphogenetic protein receptor 2; CDK—cyclin-dependent kinase; EGFR—endothelial growth factor receptor; Eph2A—erythropoietin-producing human hepatocellular type 2A receptor; ER—estrogen receptor; ErbB2/HER2—human epidermal growth factor receptor-2; ET-1—endothelin type 1; FGFR—fibroblast growth factor receptor; FLT3—FMS-like tyrosine kinase 3; HIF1a—hypoxia inducible factor 1 subunit alpha; IL-10—interleukin type 10; INF-γ—interferon gamma; LDH—dehydrogenase lactate; LP—left pneumonectomy; MAPK—mitogen-activated protein kinase; MCP-1—monocyte chemoattractant protein-1; MHC—major histocompatibility complex; mPAP—mean pulmonary artery pressure; NF-κB—nuclear factor kappa-light-chain-enhancer of activated B cells; NK—natural killer; NO—nitric oxide; PAH—pulmonary arterial hypertension; PAI-1—plasminogen activator inhibitor; PDGFR—platelet-derived growth factor receptor; ROS—reactive oxygen species; RV—right ventricle; RVH—right ventricle hypertrophy; RVSP—right ventricle systolic pressure; SAPK/JNK—stress-activated protein kinase/c-Jun NH(2)-terminal kinase; SMA—smooth muscle alpha-actin; TGF—transforming growth factor beta; TNF-α—tumor necrosis factor alpha; VEGFR—vascular endothelial growth factor receptor.

Figure 3 summarizes the effect of particular small-molecule protein kinases and their inhibitors on the PAH microenvironment, including such molecular hallmarks as inflammation, apoptosis, proliferation, or mitochondrial function.

#### 2.3.3. The Potential Determinants of Inter-Study Heterogeneity

The effect of a particular candidate agent on PH prevention (reversal) is influenced by numerous items related to individual experimental protocols (Table 2). The final result is strongly influenced by the procedure chosen for PH induction, with MCT-based models demonstrating more pronounced alleviation of the disease in treatment groups. The choice of animal species had a lesser effect, with more substantial improvement in rats receiving a particular treatment. The final result, i.e., drug efficacy, could also be affected by regimen, with BCR-ABL blockers demonstrating more pronounced effects in late therapeutic models than early preventive ones, and route of administration, with inhalation causing more significant normalization of PH, as well as by dose size and some aspects of methodological quality. In the latter case, the molecules tested in protocols where the researcher was blinded had a less pronounced impact on the normalization of PH than non-blinded protocols.

Some substances targeting the same kinases demonstrated significant discrepancies in their activity (Table 2).

#### 2.3.4. Animal Survival

**Figure 4:**
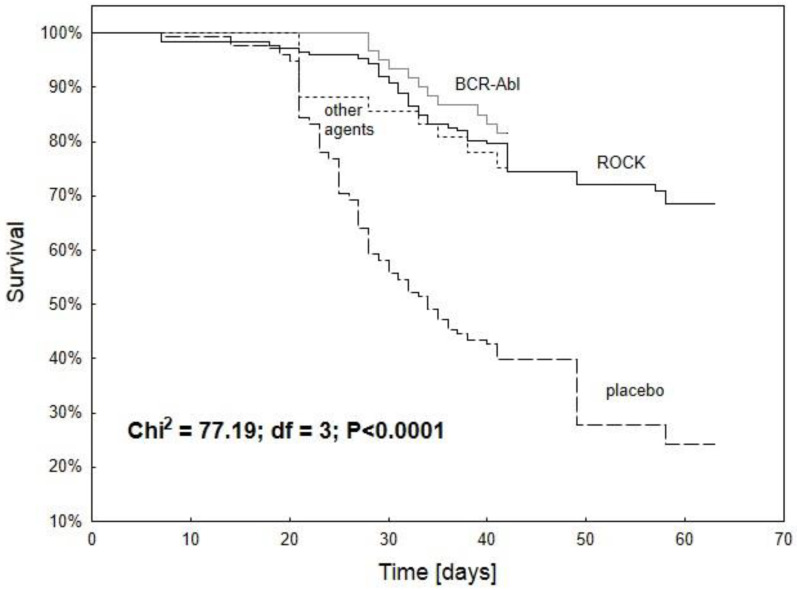
Kaplan–Meier survival curve of the overall survival of animals with monocrotaline-induced PH treated with agents targeting different kinase families (n = 461 animals). The placebo group was exposed to monocrotaline and saline. The treatment group received BCR-ABL blocker (imatinib at 1, 10, or 50 mg/kg bw), ROCK inhibitor (fasudil hydrochloride at 30 and 100 mg/kg bw), or other agents: suramin (10 mg/kg bw), BIBF1000 (50 mg/kg bw), masitinib (50 mg/kg bw), PKI166 (50 mg/kg bw), and TGFBRII-Fc (5 mg/kg bw). All substances significantly improved animal survival as compared to placebo (Chi2 = 77.19; df = 3; *p* < 0.0001). No differences were found between individual therapeutic subgroups (Chi2 = 4.87; df = 2; *p* = 0.09) [13,16,26,28,43,52,66,84,103].

In general, treatment with various small-molecule protein kinase inhibitors such as imatinib (BCR-ABL) and fasudil hydrochloride (ROCK), as well as suramin (PDGFR, FGFR, EGFR), BIBF1000 (FGFR), masitinib (PDFGR, pERK), PKI166 (EGFR), and TGFBRII-Fc, resulted in significantly improved survival (*p* < 0.0001) compared to placebo. All animals had PH due to monocrotaline injection. No significant differences were found between agents targeting different protein kinases (*p* > 0.05). Nevertheless, fasudil demonstrated more than 70% animal survival over a longer experimental period (56 to 63 days) than other molecules that were tested during shorter intervals (Figure 4).

### 2.4. Clinical Studies

Table 3 and Table S5 summarize the clinical trials assessing the efficacy and safety of small-molecule protein kinase inhibitors. Ongoing trials in phases 2 and 3 have been launched mainly to examine inhaled seralutinib and imatinib. The primary outcomes include alterations in hemodynamics (pulmonary vascular resistance, PVR) and six-minute walk distance (6MWD), the latter being a simple and valid reflection of exercise capacity. In the TORREY phase 2b study, 86 adults receiving PAH background therapy and randomly assigned to seralutinib or placebo demonstrated significantly decreased PVR after 24 weeks [106,107]. Recruitment is currently underway for the PROSERA phase 3 trial with seralutinib given by inhalation for up to 48 weeks [123]. The IMPAHCT study (NCT05036135) was a randomized, double-blind study with continuous recruitment and dose selection; phase 2b aimed to compare the effects of low, medium, and high doses of inhaled imatinib (AV-101) with placebo, with the dose demonstrating optimal efficacy and safety profile being selected for phase 3 [120]. Results from IMPAHCT demonstrated that while inhaled imatinib was well tolerated, it did not prove to be efficacious at any of the doses of AV-101 evaluated in the study, and therefore a long-term extension study of AV-101 in subjects with PAH who completed the IMPAHCT study (IMPAHCT-FUL) was terminated by the sponsor very recently (NCT05557942) [121].

## 3. Discussion

The current paper summarizes the findings of 93 preclinical investigations and 20 clinical studies, in which 51 different substances, small-molecule protein kinase inhibitors, were evaluated according to their potential efficacy and safety in PH. This is the first study that quantitatively compares the hemodynamic, echocardiographic, and histomorphometric data from preclinical trials and identifies the molecules that exert the most comprehensive effects on PH. The authors discuss animal survival and the role of individual therapies in the PAH microenvironment, including such molecular hallmarks as inflammation, apoptosis, proliferation, or mitochondrial function. The paper also provides a brief summary of clinical studies on the overviewed substances and PAH and explores the potential errors occurring when translating results from animal experiments to humans.

Most of the reviewed molecules have been approved for the management of neoplasms, and a few have been indicated for the treatment of non-neoplastic diseases. The latter include FDA or non-FDA registrations for prophylaxis of organ rejection in patients receiving renal transplants (sirolimus), graft-versus-host disease (ruxolitinib), idiopathic pulmonary fibrosis (nintedanib), and cerebral vasospasm (fasudil) [126]. Fasudil was also designated as an orphan medicine for the treatment of amyotrophic lateral sclerosis in the European Union in June 2023 [127]. Although many of these molecules are multikinase inhibitors, for the purposes of current analyses, they were classified into subgroups based on their primary target, i.e., kinase family, such as inhibitors of receptor protein-tyrosine kinase (ALK, FGFR, VEGFR), blockers of non-receptor protein-tyrosine kinases (Janus kinase—Jak1/2, BCR-ABL), protein-tyrosine kinase Abl (BCR-ABL) and protein-serine/threonine kinases (RhoA kinase—ROCK).

The present study places special emphasis on molecules whose advantageous effect on PH observed in preclinical experiments was further confirmed by normalization of molecular hallmarks and/or substantial improvements in healthy subjects/patients through phase 1–3 clinical trials. They mostly included female and male adult subjects with symptomatic PAH (II–V FC-WHO). The study participants met the criteria for one of the following categories of group 1 pulmonary hypertension: IPAH, HPAH, CTD-PAH, PAH after repair of congenital systemic to pulmonary shunt, or PAH associated with anorexigens or other drugs. The subgroups were too small to allow robust statistical comparisons between patients with CTD-PAH or repaired congenital heart disease; similarly, they did not provide any insight into improvements in hemodynamic parameters or functional capacity among IPAH or HPAH subjects [9].

### 3.1. BCR-ABL Tyrosine Kinase Inhibitors

One fifth of all interventions for PH analyzed in the current paper concerned the BCR-ABL class. The most frequently studied (15.3% interventions) is imatinib (first generation), as well as bosutinib, dasatinib, and nilotinib (second generation); all have been approved by the FDA for frontline therapy of chronic myelogenous leukemias. Ponatinib (third generation) has been registered for resistant disease due to T315I mutation or after failure with at least two other protein-tyrosine kinase inhibitors [128]. Imatinib, nilotinib, and ponatinib bind to the catalytic site of BCR-ABL in place of ATP and maintain its inactive form, thus inhibiting cell proliferation and promoting apoptosis. Dasatinib and bosutinib block the active form of BCR-ABL; they have a higher potency than imatinib and could be active on the majority of BCR-ABL-resistant cells [129].

These molecules have been found to significantly differ in their possible efficacy in PH [130]. In experimental studies, imatinib alleviated (reversed) hemodynamic and hypertrophic alterations in the right ventricle and pulmonary arteries. These effects were confirmed at the molecular level by the normalization of collagen deposition, proliferation and macrophage accumulation, and the elevation of anti-inflammatory interleukins (TNF-α, IL-10) and vasodilators such as nitric oxide [19,74]. Ponatininb demonstrated less efficacy in PH management than imatinib and nilotinib, while dasatinib promoted disease development: RV function and structure worsened, accompanied by decreased NO and increased ROS generation [94]. Moreover, imatinib more pronouncedly decreased systemic blood pressure and, like dasatinib, did not improve cardiac failure, indicated by cardiac output.

Targeted TKI therapies have revolutionized treatment for acute lymphoblastic leukemias (ALLs) and chronic myelogenous leukemias (CMLs). Recent clinical studies have provided a further insight into the negative impact of BCR-ABL TKIs, with the cardiotoxicities being identified as on- and off-target effects [129]. The most common adverse effects are pleural effusions, which are particularly associated with dasatinib and bosutinib. Dasatinib can also promote hepatobiliary disorders, hemorrhage, and PAH; the latter has been recognized as an uncommon, but serious (fatal), complication in patients with CML [128]. Dasatinib itself is believed to exert its toxic influence via inflammation of PASMCs and impaired immune system. Nilotinib and ponatinib can induce metabolism and nutrition disorders as well as vascular complications (hypertension, ischemic heart disease, ischemic cerebrovascular events) [10]. Cardiopulmonary toxicities may be caused by intense pulmonary arterial vasoconstriction and are often reversible after drug withdrawal. Although bosutinib, ponatinib, and milotinib have limited evidence in PAH [129], patients who are switched to bosutinib or ponatinib should be closely monitored in case of recurrence of the disease [128]. These unexplained respiratory complications should prompt further diagnostic evaluations (e.g., regular echocardiograms performed on patients receiving TKIs) [129]; and some strategies for surveillance and management of drug-induced pulmonary hypertension have been presented [131]. The gold standard for treatment in chronic leukemias, and the most promising agent for PH treatment among the BCR-ABL group, is imatinib. Treatment has been associated with improved exercise capacity, according to the six-minute walk test, and right ventricle hemodynamics, as reported in the IMPRES phase 3 randomized placebo-controlled trial [9]. These advantages have been associated with hemorrhagic complications with subdural hematoma, dyspnea, and syncope. As discussed above, PAH is more closely associated with dasatinib than imatinib. However, due to its unacceptable safety profile and high rates of discontinuation, imatinib is not a suitable treatment for PAH. Interestingly, the current analysis of survival curves for imatinib did not reveal any significant differences in animal mortality as compared to other small-molecule protein kinase inhibitors; in addition, while some authors presented survival curves, most papers did not report whether the animals were lost throughout the experiments. Most studies with BCR-ABL TKIs were relatively short, i.e., up to 42 days. More than 40% of papers did not provide accurate numbers of the animals in each group at the beginning and end of the experiment, making it impossible to determine any loss and the cause. As such, the data about survival of animals treated with BCR-ABL are inconclusive.

Most importantly, imatinib and other BCR-ABL TKIs are actually multikinase inhibitors, with a wide spectrum of off-target inhibition of PDGF, FGF, or mast/stem cell growth factor (c-KIT) receptors [132].

### 3.2. Platelet-Derived Growth Factor Receptor Blockers

The platelet-dependent serum growth factor stimulates the proliferation of fibroblasts and arterial smooth muscle cells and regulates embryonic development, particularly vessel and organ formation [133]. Activation of the PDGF/PDGFR pathway has been demonstrated in patients with pulmonary hypertension, where PDGF isoforms could control vascular cell migration, proliferation, and survival through binding to their PDGFRs [84].

In line with these observations, the preclinical studies included in this review were based on animal models known to activate the PDGF pathway in response to PH stimuli, such as monocrotaline or hypoxic conditions [35,36,37,38,39,40,41,42,43,44,45]. Smooth muscle cells (SMCs) play a key role in PH-associated vascular remodeling, particularly due to pulmonary vascular proliferation and muscularization. PASMCs mostly express PDGFRβ, which induces SMC proliferation by activating the mitogen-activated protein kinase (MAPK), Akt, and JAK and STAT1/3 pathways [134]. PDGFRβ activation was also revealed to provoke pulmonary artery SMC contraction via mechanisms associated with prostaglandins, calcium and cAMP, MAPK, or PI3K/AKT/mTOR signaling and actin remodeling [135].

Recent experimental studies on PH have identified a number of molecules that can non-specifically target the PDGF/PDGFR pathway. These include the BCR-ABL blockers dasatinib, imatinib, nilotinib, and ponatinib, the VEGFR and FGFR blockers BIBF1000, dovitinib, regorafenib, and sunitinib, the mTOR inhibitor sirolimus, and the combined tyrosine and the serine/threonine kinase inhibitor sorafenib. The pathway is also modulated by seralutinib, which targets PDGF/c-KIT/BMP receptor type 2 (BMPR2) signaling [8]. Interestingly, the present analyses did not find that these molecules more effectively reversed or prevented PA remodeling or RV hypertrophy compared to the substances that do not target the PDGF pathway but act on other ones. Recent studies on PH and similar diseases have focused on therapies that specifically target only one member of the PDGFR/PDGF family; these include PDGFR/PDGF blocking antibodies or anti-PDGFR (anti-PDGF) oligonucleotides (siRNA, miRNA, DNA) [10], and this could be another promising strategy targeting this signaling pathway.

### 3.3. Endothelial Growth Factor Receptor Blockers

In recent years, the EGFR/ErbB family has become the target for several anti-cancer therapeutics. The EGFR is a membrane-bound receptor TK within the subfamily of HER1/EGFR/ERBB1, HER2/NEU/ERBB2, HER3/ERBB3, and HER4/ERBB4 receptors [136]. Gefitinib, erlotinib, and lapatinib are EGF inhibitors clinically approved by the FDA for the treatment of advanced non-small cell lung cancer (NSCLC); icotinib has been registered for such use in Asia [11].

The current synthesis of preclinical studies demonstrates that treatment with an individual EGFR inhibitor reduced RVSP and/or mPAP and right heart hypertrophy in rats with induced PH. Measurements of medial wall thickness and the degree of muscularization of small peripheral pulmonary arteries indicated improvements in pulmonary vascular remodeling. The molecules targeting the EGF receptor demonstrated good efficacy among small-molecule protein kinase inhibitors used in experimental protocols comprising 200 animals (8% of interventions). Further molecular studies on animal lungs demonstrated that gefitinib, erlotinib, and lapatinib inhibited PASMC proliferation in rats treated with EGF, but without substantial alterations in pulmonary EGFR expression. In vitro human studies revealed no significant changes in EGFR expression in lung tissues from patients with idiopathic PAH [25]. Consequently, it remains unclear whether targeting EGF signaling might be a promising therapeutic approach for PH, and no clinical trials have been designed for EGFR blockers or PAH.

### 3.4. Vascular Endothelial Growth Factor Receptor Blockers

VEGF-A has been recognized as a major angiogenic growth factor. Also, the activation of VEGFR2 receptors on vascular endothelial cells following tyrosine phosphorylation results in the initiation of a number of downstream signaling events that determine endothelial cell survival, proliferation, migration, and permeability [137]. Both experimental and clinical observations have confirmed that VEGF-A and its receptors demonstrate increased expression in pulmonary hypertension [138]; however, the role of this signaling pathway in RV hemodynamics and hypertrophy is still poorly understood. For example, inhibition of VEGF-A/VEGFR2 signaling by the tyrosine–kinase inhibitor Sugen5416 (SU5416) has been shown to aggravate, not diminish, pulmonary vasculopathy triggered by chronic hypoxia. This has given rise to the in vivo model of Sugen/hypoxia-induced PH for experimental studies [139]. A very recent study demonstrated that abolishing VEGFR2 Y949 signaling by Vefgr2Y949F point mutation may alleviate PH-related lesions, as well as PA muscularization, RV hypertrophy, and elevated RVSP, without inhibiting all the effects of VEGFR2 signaling [137]. The current synthesis of preclinical studies on PH seems to support this hypothesis; selective but entire inhibition of VEGFR2 by cabozantinib tended to exacerbate pulmonary hypertension. However, this was not the case for other, non-selective VEFGR blockers e.g., sunitinib, sorafenib, or regorafenib. These molecules act as multikinase inhibitors and were found to reverse (alleviate) PH as a result of targeting VEGFR and other receptors, such as FGFR (BIBF1000), PDGFR-β or FGFR1 (regorafenib), PDGFR-β or c-KIT (sunitinib), b-RAF, c-KIT, or PDGFR-β (sorafenib). For the purposes of the current analysis, they were classified together into a subgroup with VEGFR as their primary target [11].

Among these agents, sorafenib was found to most effectively alleviate PH; however, it also resulted in higher systemic blood pressure (≈16 mmHg) than the other TKIs. It is believed that sorafenib exerted a positive effect on pulmonary hypertension via the inhibition of pulmonary VEGFR2 and VEGF-A expression and the suppression of VEGF-A protein [140]. Sorafenib is already registered for the treatment of hepatocellular and renal carcinomas. A phase 1b single-center, open-label study found that 200 mg doses might be safely added to the target therapy for PAH (prostacyclins), but without any pronounced improvement in pulmonary vascular resistance or exercise capacity (six-minute walk test) [112].

### 3.5. Mammalian Target of Rapamycin Kinase Blockers

Mammalian target of rapamycin (mTOR) is a serine/threonine (Ser/Thr) protein kinase. It forms two unique complexes, mTORC1 and mTORC2, and has been established as one of the major molecular targets for anti-proliferative therapies [141]. Sirolimus (rapamycin), everolimus, and temsirolimus have been approved by the FDA for the treatment of HER2-negative breast cancer, pancreatic neuroendocrine tumors, renal cell carcinomas, or angiomyolipomas. Rapamycin has also been registered for immune suppression after organ transplantation [11]. Previous experimental trials found that mTOR signaling (mTORC1) might play a key regulatory function in cardiovascular physiology and pathology including left cardiac remodeling and systolic dysfunction [142].

mTOR can stimulate a number of PH-related pathways, such as HIF-1α, Notch, nuclear factor kappa B (NF-κB), signal transducer and activator of transcription 3 (STAT3), and peroxisome proliferator-activated receptor gamma (PPARγ). AKT/mTOR has also been recognized to play an important role in cell proliferation induced by various growth factors, including PDGF or FGF2 [143]; interestingly, Goncharov et al. (2014) found both mTORC1 and C2 to mediate vascular cell proliferation in human PH [144].

As such, further studies are merited on the potential of mTOR-therapeutic targets for the treatment of pulmonary hypertension. Present analysis demonstrates that sirolimus and its derivatives, everolimus and temsirolimus, either reversed or prevented hemodynamic issues, as well as PA remodeling and RV hypertrophy. The potential of rapamycin in PH is still under investigation. A phase 1/1b study is currently assessing the maximum-tolerated dose and safety profile of nab-sirolimus in patients with severe pulmonary arterial hypertension (NCT02587325); as yet, the results have not been posted [125].

### 3.6. Rho-Associated Kinase Inhibitors

Rho-associated kinases (ROCKs) act as downstream effectors of the small GTP-binding protein Rho. They belong to the serine/threonine cAMP-dependent protein kinase group, and can exist in two isoforms, ROCK1 and ROCK2, with the latter being found predominantly in heart, lung, smooth muscle, and brain. ROCK1/2 regulate the activity of muscle myosin regulatory light chain protein [145]. As patients with idiopathic PAH (IPAH) demonstrate higher ROCK activity in the lungs and PASMCs [146], there has been growing interest in experimental research on ROCK inhibitors in PH. Consequently, one fifth of all interventions included in the current paper concern the ROCK pathway; the preclinical trials associated with ROCK have been performed in a variety of animal models exposed to monocrotaline, bleomycin, chronic hypoxia (combined with VEGFR inhibitor), or aortic banding.

Among a wide spectrum of protein kinase inhibitors, the selective ROCK inhibitor fasudil hydrochloride manifested a considerably greater effect on reversal (prevention) of pulmonary artery remodeling and RV hypertrophy; it also demonstrated good drug tolerability and >70% animal survival within a 56- to 63-day experimental period according to Kaplan–Meier curves. It was found that fasudil lowered the levels of the vasoconstrictor endothelin type 1 and the pro-inflammatory interleukin IL-17 and reduced lung fibrosis, oxidative stress, and proliferation. It also increased the levels of NO and anti-inflammatory interleukin IL-10 [105]. These promising results have been only partially confirmed in clinical observations. While the acute administration of fasudil was found to effectively alleviate mPAP and PVR [109,114,115], clinical evidence is lacking about the long-term effects of chronic treatment, including improvements in exercise and survival.

### 3.7. Other Targets for Small Protein Kinase Inhibitors

A few recent experimental trials have identified other small-molecule protein kinase inhibitors as potential therapeutic options for PH management. The Janus kinase/signal transduction and activator of transcription (JAK/STAT) pathway has been known to play a significant role in the development of PH particularly through the activation of JAK2 and STAT3 proteins. Signaling cross-talks between JAK/STAT and other pathways (e.g., TGFβ, MAPK, Notch, PI3K/AKT/mTOR, NF-κB, etc.) can promote pluripotency and differentiation, immune dysregulation, and tumorigenesis [146]. As a result, the JAK/STAT pathway mediates signal transduction induced by various cytokines, interferons, and growth factors (e.g., TGF-β, PDGF, interleukin IL-6, or granulocyte colony-stimulating growth factor) [47,147], and protein inhibitor of activated STAT (PIAS), SOCS/CIS family members, and protein tyrosine phosphatases (PTPs) can negatively regulate JAK/STAT signal transduction [148]. Suppressing these factors has anti-inflammatory effects and can impair the synthesis of collagen and inhibit SMC and fibroblast proliferation. These features can be important when considering the pathogenesis of inflammatory and autoimmune diseases such as rheumatoid arthritis, psoriasis, ulcerative colitis, and graft-versus-host disease [126,149]. The involvement of the JAK/STAT cascade in inflammation and fibrosis in lung tissue has driven studies on the use of the JAK1 and JAK2 inhibitor ruxolitinib in chronic thromboembolic pulmonary hypertension [101]. PASMCs from patients with IPAH were found to demonstrate increased Janus kinase 2 expression, prompting further preclinical evaluations. Recent investigations initially revealed a significant increase in cardiac efficiency, indicated by cardiac output, accompanied by a slight improvement in RV hypertrophy or PA muscularization. Molecular studies indicate a reduction in proliferation processes and decreased levels of pro-inflammatory IL-1, IL-6, and IL-10 [101].

As FGF2 overproduction has been noted in the human vascular endothelium in idiopathic PAH, it is possible that fibroblast growth factors might represent potential targets for disease management [150]; however, dovitinib, infigratinib, or nintedanib treatment did not appear to substantially reverse PH manifestations. The opposite was observed for two other novel multikinase inhibitors, regorafenib and BIBF1000, which demonstrated non-specific activity against FGF and VEGF receptors. Other studies have been performed to evaluate the potential advantages of FGFR blockers in pulmonary diseases such as pulmonary fibrosis, asthma, lung injury, or chronic obstructive pulmonary disease [150].

Recent findings indicate increased expression of activin type-I and type-II receptors in the vascular cells of explanted lungs obtained from patients with PAH, defining another potential target for PH treatment [151]. The ALK family (ALK2, ALK4, ALK5, and ALK7) can transmit signals via a number of pathways including Smad, often accompanied by impaired bone morphogenetic protein, and TGF-β, associated with endothelial proliferation, migration, and lung remodeling [152]. Further trials are needed to confirm whether targeting the ALK pathway can reverse PH; present data indicate that inhibition of the activin receptor-like kinase pathway might result in slight reversal ([S]-crizotinib, IN-1233, K02288) or even progression ([R]-crizotinib) of pulmonary hypertension.

Recent findings also highlight the potential of using multiple kinase signaling networks as targets for PAH management. For example, CSF1R, PDGFR, c-KIT, and BMPR2 kinase signaling pathways have each been implicated in the pathogenesis of PAH, but they can also interact in complex networks to exert downstream biological effects. As described by Galkin et al. (2022) [31], activated CSF1R-positive macrophages and c-KIT-positive pulmonary artery endothelial cells can secrete PDGF, which binds to PDGFRs on PASMCs and fibroblasts; in turn, PDGFR activation induces several signaling cascades, viz. PI3K, AKT, STAT, and ERK. Such activation increases PASMC and fibroblast proliferation and enhances the medial hypertrophy of pulmonary arterioles and perivascular fibrosis. PDGFR stimulation can also downregulate BMPR2, thus further exacerbating proliferation.

The activation of c-KIT in mast cells can also trigger abnormal proliferation and inflammation, as well as pulmonary vascular remodeling [31]. Seralutinib as a small-molecule, multikinase blocker that inhibits PDGFRα/β, CSF1R, and c-KIT and increases BMPR2 levels. Its positive effects on signaling pathways have been evaluated in preclinical and clinical trials, and the initial results appear very promising [8,106,107].

### 3.8. The Potential Gaps in Translation of Preclinical Results

Numerous small-molecule protein kinase inhibitors have been studied in preclinical protocols for their potential efficacy in pulmonary hypertension. Despite generally promising results, there is still insufficient clinical evidence for introducing such a novel therapeutic pathway in addition to the existing nitric oxide, endothelin, and prostacyclin approaches. Most existing clinical trials have failed to confirm whether these molecules have a satisfactory safety profile (imatinib, nilotinib) or demonstrate any long-term improvement in exercise capacity or RV hemodynamics (fasudil, sorafenib). Like the agents targeting the EGF family, some experimental investigations were not supported by observations confirming the contribution of the targeted pathway to disease development in humans; this could potentially increase the risk of unsuccessful translation of preclinical experiments to human studies.

In addition, PAH studies lack a standardized experimental approach. The animal model should reflect the clinical condition associated with the clinical classification of the disease. This also prevents the successful identification of candidate drugs for further clinical evaluations. PAH has a diverse and multifactorial etiology: it can be idiopathic, inherited, or induced by drugs (toxins). It can also be complicated with other diseases. For example, it is most commonly associated with connective tissue disorders (CTDs) including systemic sclerosis (SSc), which accounts for seventy-five percent of PAH-CTD cases [153]. Vasculopathy, activation of the cellular immune system, inflammation, and fibrosis are all recognized as pathogenetic hallmarks of SSc [154], and this should be reflected by the animal models selected for preclinical experiments on PAH-SSc. New therapeutic options and their long-term effects on PAH-SSc are being evaluated in several trials [155]. The current review includes only three studies which used a bleomycin model for inflammation-driven skin lesions to assess the efficacy of fasudil hydrochloride, ponatinib, or JSI-124 on idiopathic pulmonary fibrosis and pulmonary hypertension [20,46,67]. Only one study, on nilotinib, employed a model intended to reflect PAH associated with systemic sclerosis: Fos-related antigen-2 (Fra-2) transgenic (tg) mice [62]. Very few studies have examined the effect of the PAH-CTD approach on the final outcome compared to common rodent models. In addition, the effects of TGFβ signaling on PAH have been assessed in animals exposed to monocrotaline or chronic hypoxia, but not kinase-deficient TGFβRII mice [103]. This is also a limitation of the current study.

The inability of animal models to completely reflect human disease has been widely discussed [156]. In the studies in the current review, in addition to monocrotaline (clinical group 1, PAH), animals could be exposed to chronic hypoxia (clinical group 3, PH associated with lung diseases and/or hypoxia) or aortic banding (clinical group 2, PH associated with left heart disease). Only 25% of papers included “head-to-head” analyses, where two or more animal models (e.g., chronic hypoxia and monocrotaline) were used to assess the potential advantages of an individual agent. Even fewer, i.e., 11.8%, concerned such “head-to-head” comparisons between two regimens, e.g., early and late protocols. Such comparisons would strengthen conclusions about the preclinical effectiveness of potential candidate drugs for PAH.

As a consequence, the current analysis demonstrates the relatively high heterogeneity between different studies based on the same molecule. For example, experiments in which the candidate drug (BCR-ABL, EGFR, FGFR blockers) was given to monocrotaline-treated rats yielded more pronounced alleviation of PH. These phenomena also concerned aortic-banded subjects (fasudil) or animals exposed to SU5416+chronic hypoxia (VEGFR blockers). Other sources of discrepancies included animal species, with more advantageous effects observed in rats than in mice, and route of administration: for example, BCR-ABL inhibitors displayed better results when given by inhalation or intratracheally compared to intragastric or intraperitoneal administration.

Another potential determinant of such heterogeneity between preclinical studies could be their methodological quality. Most of the papers included in the current systematic review demonstrated an unclear risk of bias, mainly concerning a lack of information about the randomization procedure or allocation concealment. As discussed previously, poor reporting of the methodology applied in an individual study can negatively impact its repeatability and reproducibility. In line with previous findings, the current analysis demonstrates that blinding the researchers can impact the obtained outcome and can be a source of heterogeneity between studies. The candidate drug demonstrated significantly less activity among animals in which the hemodynamic or histomorphometric measurements were performed blind; this result also confirms that blinding might reduce the risk of the true effect of the intervention being overestimated.

### 3.9. Future Perspectives

While recent clinical evaluations have yielded unsatisfactory results, some small-molecule protein kinase inhibitors have nevertheless been of great interest for studies on PH. For example, when imatinib is administered intratracheally or as inhalation powder, being incorporated in nanoparticles, the drug might reduce the adverse effects associated with treatment. Indeed, current preclinical findings confirm it has a more pronounced effect on RV hemodynamics and vascular remodeling when targeting the lungs directly compared to intragastric administration. To date, two clinical trials have been launched to evaluate the advantages of such an innovative treatment with imatinib. While inhaled imatinib was well tolerated, it did not prove to be efficacious in doses proposed for the study, and further trials are needed.

Another therapeutic agent for PH treatment is seralutinib. This small-molecule kinase inhibitor targets PDGFRα/β, CSF1R, and c-KIT and increases BMPR2 signaling in a PDGF-dependent manner. The latter can counteract exacerbation of proliferation, as well as PASMC proliferation driven by TGFβ in the pulmonary arterioles. The molecule targets inflammatory, proliferative, and fibrotic pathways implicated in the pathology of PAH. Seralutinib was found to decrease TNF-α and NT-proBNP and increase IL-10 [8]. In the current analysis, inhaled seralutib was found to be particularly effective at reversing pulmonary vascular remodeling and improving RV hemodynamics parameters compared to other small-molecule protein kinases. The TORREY phase 2 clinical trial confirmed that inhaled seralutinib has good tolerability in healthy volunteers and PAH patients [104], and the results support the rationale for a further phase 3, randomized, double-blind, placebo-controlled study (PROSERA) (NCT05934526) [123].

Numerous pathogenic mutations of the gene encoding BMPR2, a serine/threonine kinase, have been reported to cause PAH. Its reduced expression can result in exaggerated signaling responses to TGFβ, apoptosis of endothelial cells, and abnormal proliferation of PASMCs in response to TGFβ and BMP ligands [157]. Thus, apart from selaratunib that non-specifically targeted this pathway, selective BMPR kinase inhibitors have been recognized as interesting therapeutic options to normalize BMPR signaling [158]. Several agents have been found to target different levels within the BMP signaling cascade, including the Src kinase inhibitor saracatinib. The molecule has been tested so far in experimental models of idiopathic pulmonary fibrosis, as well as in human subjects with Alzheimer’s disease or with various types of cancer [158].

## 4. Materials and Methods

The PUBMED, EBSCO (MEDLINE), and clinicaltrials.gov databases were searched for preclinical (full-text papers) and clinical studies (abstracts, full-text papers) regarding the ability of therapeutic agents targeting kinase families to reverse pulmonary hypertension. The search included preclinical studies addressing a wide spectrum of rodent models and experimental protocols on PH. They must have reported alterations in at least one PH-related parameter due to chronic exposure to an individual protein kinase inhibitor. All papers were published from January 2001 to October 2023.

The search (secondary aim) also included prospective clinical studies (recruiting, ongoing, completed, or terminated) including adult healthy participants or adult subjects with PAH, with primary outcomes defined as safety or efficacy (such as improvements in 6MWD, hemodynamic parameters, or survival) of an individual small-molecule protein kinase inhibitor. The search strategy is presented in more detail in the Supplementary Materials.

To allow further quantitative assessments, the following preclinical data were recorded, according to PICO, as described previously [159]: PH-related lesions associated with hemodynamic parameters, such as mean pulmonary artery pressure—mPAP, systolic right ventricle pressure—RVSP, cardiac output—CO, cardiac index—CI, systemic blood pressure—BP. In addition, right ventricle hypertrophy (RVH), the degree of pulmonary artery (PA) remodeling, and the number of animals surviving the experiment were recorded [159]. The exclusion criteria for preclinical protocols were as follows: (a) review articles, (b) studies using in vitro, ex vivo models, (c) studies using animals other than rodents, (d) protocols intended to evoke cardiotoxicity or non-PH disease, (e) protocols that did not report PH-related parameters, (f) studies not addressing effects of therapeutic agents on PH, (g) studies reporting effects of gene therapy. The exclusion criteria also concerned clinical protocols where an individual tyrosine kinase inhibitor was investigated in a non-PH indication.

The electronic databases were searched by two independent reviewers (MJ-S, PG), who extracted the relevant descriptive and numerical data and assessed the risk of bias using the SYstematic Review Centre for Laboratory animal Experimentation (SYRCLE) tool [160]. Publication bias was assessed by performing an Egger’s weighted regression test and the Duval and Tweedie “trim and fill” procedure. Sensitivity analysis—with the leave-one-out method—was used to assess differences between subgroups and to verify whether the result in individual protocols might be robust. Subgroup analysis was used to identify the presence and extent of statistical heterogeneity. Data analysis was performed as described previously [161]. For details, see the Supplementary Materials. The systematic review and analyses were performed in compliance with PRISMA 2020 [162].

## 5. Conclusions

There is still a need for targeted therapies in PAH that reverse the proliferative pulmonary vascular processes and improve RV function for better long-term prognosis. Preclinical observations have found that most of the reviewed small-molecule protein kinase inhibitors are able to control migration, proliferation, and survival in pulmonary smooth muscle cells by targeting a wide range of intracellular signaling pathways. Recent studies have also focused on molecules that can influence the multiple networks of kinase signaling and their downstream effectors, such as PDGFRα/β/CSF1R/c-KIT/BMPR2. Most studies now focus on achieving a satisfactory safety profile for the drug and have evaluated innovative therapeutic approaches such as direct intratracheal drug administration and oligodeoxynucleotide, siRNA, or miRNA delivery to act specifically on individual protein kinase pathways. However, any drug molecules selected for clinical evaluations should first be tested using standardized animal models and appropriate experimental conditions, with high-quality reporting. This can successfully reduce inter-study heterogeneity, improve repeatability and reproducibility, and could provide more appropriate identification of novel promising small-molecule protein kinase inhibitors for PAH. The most important take home-messages are summarized in Table 4. 

## Figures and Tables

**Table 1:** An overview of efficacy of small-molecule protein kinase inhibitors in preclinical trials on PH and their registry status.

Primary Target	Drug	Model	Effect #	Other Targets	Registration in Humans (Date of First Approval)	References
ALK	(S)-Crizotinib (R)-Crizotinib IN-1233 K02288	MCT; SU+CH SU+CH MCT; CH CH	↓RVSP; ↓mPAP; ↔RVH; ↑CO (CI) ↑RVSP; ↑mPAP; ↔remodel; ↓CO (CI) ↓RVSP; ↔RVH ↓RVSP; ↔RVH; ↔remodel	- ROS1 - -	NSCLC (2011) NSCLC (2011) - -	[18,48,60,92,126]
BCR-ABL	Dasatinib Imatinib Nilotinib Ponatinib	MCT MCT; CH Fra-2 Tg; MCT BLEO	↓RVSP; ↔mPAP; ↔RVH; ↔remodel; ↔CO (CI) ↓RVSP ^a^; ↓mPAP ^a^; ↓RVH ^b^; ↓remodel; ↓BP; ↔CO (CI) ↓RVSP; ↓RVH; ↓remodel; ↔CO (CI) ↓RVSP; ↓RVH	c-Kit; PDGFR-α/β c-Kit; PDGFR-α/β c-Kit; PDGFR-α/β -	Ph+ CML; ALL (2006) Ph+ CML; ALL (2001); GIST (2022) Ph+ CML (2007) ALL; CML (2012)	[15,19,24,34,35,44,45,46,49,54,65,74,76,84,85,90,98,126]
EGFR	Erlotinib Gefitinib Icotinib Lapatinib PKI166	MCT; CH MCT; CH MCT MCT; CH MCT	↓RVSP; ↓RVH; ↓remodel; ↔CO (CI); ↓BP *^p^*^=0.053^ ↓RVSP;↓mPAP; ↓RVH; ↓remodel; ↑CO (CI); ↔BP ↓RVSP; ↓RVH; ↓remodel ↓RVSP; ↓RVH; ↓remodel; ↑CO (CI); ↔BP ↓mPAP; ↓RVH; ↑CO (CI); ↔BP	- - - - -	NSCLC; PC (2004) NSCLC (2003) NSCLC ** (2012) HER2-positive BC (2007) -	[25,38,66,72,78,90,126]
FGFR	BIBF1000 Dovitinib Infigratinib Nintedanib	MCT; PAB MCT MCT SU+CH	↓RVSP; ↓mPAP; ↓RVH; ↑CO (CI); ↑BP ↓mPAP; ↓RVH ↑RVSP; ↓RVH ↔RVSP; ↔RVH; ↔CO (CI); ↔BP	VEGFR; PDGFR VEGFR; PDGFR TGF-β1 -	- - Cholangiocarcinomas (2021) IPF (2014)	[16,27,29,79,82,83,89,94,126]
FKBP12/mTOR	Everolimus Sirolimus PP1	MCT MCT; CH; shunt; LP+MCT SU+CH	↓RVSP ↓RVSP; ↓mPAP; ↓RVH; ↓remodel; ↑CO (CI); ↔BP ↓RVSP; ↓RVH	- - - Akt/mTOR/HIF-1	HER2-positive BC (2009) Kidney transplants (1999); LAM (2015); PEComa (2021) -	[17,39,40,58,61,63,64,71,75,77,85,93,104,126]
JAK-STAT	Ruxolitinib JSI-124	MCT; CH BLEO	↓RVSP; ↔RVH; ↔remodel; ↑CO (CI); ↔BP ↓RVH	- -	Myelofibrosis (2011); polycythemia vera (2014); GVHD (2019); atopic dermatitis (2021) -	[47,67,101,126]
ROCK	Fasudil hydrochloride Fasudil dichloroacetate Y27632 KD02	MCT; CH; SU+CH; BLEO; AB; shunt SU+CH CH MCT	↓mPAP; ↓RVH; ↓remodel; ↔BP ↓mPAP; ↓RVH; ↓remodel; ↔BP ↓RVSP ↓RVSP	- - - -	Cerebral vasospasm * (1995) - - -	[13,14,20,26,28,32,33,36,37,45,55,57,59,69,70,80,81,87,88,95,99,100,102,126]
TGF	TGFBRII-Fc	MCT; CH; SU+CH	↓mPAP; ↓RVH; ↓remodel; ↔BP	-	-	[103]
VEGFR	Cabozatinib Regorafenib Sorafenib Sunitinib Toceranib	SU+CH MCT; CH MCT; PAB; CH PAB MCT	↔RVH ↔RVSP; ↓RVH; ↔CO (CI); ↔BP ↓RVSP; ↓mPAP; ↓RVH; ↓remodel; ↑CO(CI); ↑BP ↓RVSP; ↓RVH; ↓remodel; ↑CO (CI) ↓RVSP; ↓RVH; ↓remodel	- FGFR 1; PDGFR-β PDGFR-β; c-Kit PDGFR-β; c-Kit; b-RAF -	TC; RCC; HCC (2012) Colorectal cancer; GIST; HCC (2012) RCC; HCC (2007) GIST; RCC (2006) -	[30,49,50,51,52,54,68,91,126]
Other/ multiple targets	BCG-3111 BI 6727 Masitinib MIF098 Palbociclib PH797804 R428 Seralutinib SB204741 Suramin	MCT SU+CH; CH MCT CH MCT PAB; CH SU+CH SU+CH LP+MCT MCT	↓RVSP; ↓RVH; ↓remodel ↓RVSP; ↓RVH; ↑CO (CI); ↔BP ↓RVH; ↓remodel ↓RVSP; ↓RVH ↓RVH; ↓remodel; ↔BP ↔RVSP ↔RVSP; ↓CO (CI) ↓RVSP; ↓mPAP; ↓RVH; ↑CO (CI) ↓RVSP; ↔CO (CI) ↓mPAP; ↓RVH	NF-κB/MAPK HIF1a PDGFR; pERK PDGFR CDK6 p38 MAPK AXL; BMPR2 PDGFR; c-Kit; BMPR2 SRC tyrosine kinase; BMPR2 PDGFR, FGFR, EGFR	- - - - ER-/HER2-positive BC (2015) - - - - -	[22,31,41,43,50,53,62,73,96,97,102,126]

For detailed information—see Table S4 (Supplementary Materials). #—only significant results (*p* < 0.05) of comparisons between at least 3 interventions per group are demonstrated; ^a^—*p* < 0.0001 for comparison between imatinib given per os and by inhalation (Q = 16.68; df = 1); ^b^—*p* < 0.0001 for comparison between imatinib given per os and by inhalation (Q = 26.34; df = 1); *—Japan; **—China; Alk—activin receptor-like kinase; ALL—acute lymphoblastic leukemia; BC—breast cancer; BLEO—bleomycin; BMPR2—bone morphogenetic protein receptor 2; BNP—brain natriuretic peptide; BP—blood pressure; CDK—cyclin-dependent kinase; CH—chronic hypoxia; CI—cardiac index; CML—chronic myelogenous leukemia; CO—cardiac output; ER—estrogen receptor; ErbB2/HER2—human epidermal growth factor receptor-2; ET-1—endothelin type 1; FGFR—fibroblast growth factor receptor; Fra-2—Fos-related antigen-2 transcription factor; GIST—gastrointestinal stromal tumor; GVHD—graft-versus-host disease; HCC—hepatocellular carcinoma; HIF1a—hypoxia inducible factor 1 subunit alpha; INF-γ—interferon gamma; IPF—idiopathic pulmonary fibrosis; LAM—lymphangioleiomyomatosis; LDH—dehydrogenase lactate; LP—left pneumonectomy; MAPK—mitogen-activated protein kinase; MCT—monocrotaline; MHC—major histocompatibility complex; mPAP—mean pulmonary artery pressure; NF-κB—nuclear factor kappa-light-chain-enhancer of activated B cells; NK—natural killer; NO—nitric oxide; NSCLC—non-small cell lung cancer; PAB—pulmonary artery banding; PAI-1—plasminogen activator inhibitor; PC—pancreatic cancer; PDGFR—platelet-derived growth factor receptor; PEComa—perivascular epithelioid cell tumor; Ph+—Philadelphia chromosome positive; RCC—renal cell carcinoma; ROS—reactive oxygen species; RV—right ventricle; RVH—right ventricle hypertrophy; RVSP—right ventricle systolic pressure; SMA—smooth muscle alpha-actin; SU—Sugen; TC—thyroid cancer; TGF—transforming growth factor; VEGFR—vascular endothelial growth factor receptor. ↓; (↑; ↔)—decrease (increase; no difference) in the parameter in the Intervention group as compared to placebo.

**Table 2:** The influence of potential therapeutic agents on PH reversal (prevention) according to their primary target and characteristics of preclinical study protocol.

Target	Drug	Effect (Intra-Group Heterogeneity)	Animal Model/Regimen/ Species	Covariates *
ALK	(S)-Crizotinib; (R)-crizotinib; IN-1233; K02288	↔HEM (Q = 126.37; df = 3; *p* < 0.0001); ↓RVH (Q = 1.49; df = 2; *p* > 0.05); ↔remodel (Q = 6.95; df = 3; *p* = 0.07); ↑CO (Q = 9.74; df = 1; *p* = 0.001)	CH; MCT; SU+CH(+Nx)	-
BCR-ABL	Dasatinib; imatinib; nilotinib; ponatinib	↓HEM (Q = 12.19; df = 3; *p* = 0.007); ↓RVH (Q = 1.99; df = 3; *p* > 0.05); ↓remodel (Q = 1.43; df = 2; *p* > 0.05)	CH; MCT; SU+CH(+Nx); BLEO; early; late; inh, ig, ip; blinded/not blinded assessment of the outcome #	Animal model (Q = 34.61; df = 3; *p* < 0.0001); regimen (Q = 7.74; df = 1; *p* = 0.0054); imatinib dose (*p* = 0.017); imatinib route of administration ** (Q = 10.22; df = 2; *p* = 0.006); blinding (Q = 18.93; df = 1; *p* < 0.0001)
EGFR	Erlotinib; gefitinib; icotinib; lapatinib; PKI166	↓HEM (Q = 3.75; df = 4; *p* > 0.05); ↓RVH (Q = 4.71; df = 4; *p* > 0.05); ↓remodel (Q = 12.29; df = 4; *p* < 0.01); ↑CO (CI) (Q = 2.25; df = 2; *p* > 0.05)	CH; MCT; mouse; rat	Animal model (Q = 62.47; df = 1; *p* < 0.0001); species (Q = 45.24; df = 1; *p* < 0.0001)
FGFR	BIBF1000; dovitinib; infigratinib; nintedanib	↓HEM (Q = 42.16; df = 3; *p* < 0.0001); ↓RVH (Q = 11.35; df = 3; *p* = 0.01); ↓remodel (Q = 46.74; df = 3; *p* < 0.0001)	MCT; SU+CH(+Nx); PAB; blinded/not blinded assessment of the outcome #	Animal model (Q = 37.57; df = 2; *p* < 0.0001); blinding (Q = 3.17; df = 1; *p* = 0.07)
JAK-STAT	Ruxolitinib	↓HEM	CH; MCT; mouse; rat	Animal model (Q = 11.45; df = 1; *p* = 0.0007); species (Q = 9.12; df = 1; *p* = 0.002)
mTOR	Everolimus; PP1; sirolimus; temsirolimus	↓HEM (Q = 3.14; df = 1; *p* = 0.08); ↓RVH (Q = 239.9; df = 3; *p* < 0.0001); ↓remodel (Q = 0.1; df = 1; *p* > 0.05)	CH; LP+MCT; MCT; SU+CH(+Nx); CA-JV shunt; SM22-5HTT+	Animal model (Q = 44.86; df = 5; *p* < 0.0001)
ROCK	Fasudil hydrochloride; fasudil dichloroacetate	↓HEM (Q = 7.66; df = 1; *p* < 0.006); ↓RVH (Q = 0.01; df = 1; *p* > 0.05); ↓remodel (Q = 1.82; df = 1; *p* > 0.05)	CH; MCT; SU+CH(+Nx); aortic banding; BLEO; shunt; mouse; rat; blinded/not blinded assessment of the outcome #	Animal model (Q = 97.74; df = 6; *p* < 0.0001); species (Q = 54.96; df = 1; *p* < 0.0001); fasudil dose (*p* < 0.0001); blinding (Q = 3.96; df = 1; *p* = 0.046)
TGF	TGFBRII-Fc	-	MCT; SU+CH(+Nx); LP+MCT; early; late	Animal model (Q = 7.63; df = 2; *p* = 0.022); regimen (Q = 9.77; df = 1; *p* = 0.0018)
VEGFR	BIBF1000; cabozantinib; regorafenib; sorafenib; sunitinib; toceranib	↓HEM (Q = 30.47; df = 4; *p* < 0.0001); ↓RVH (Q = 13.53; df = 4; *p* < 0.009); ↓remodel (Q = 126.10; df = 4; *p* < 0.0001)	CH; MCT; SU+CH(+Nx); PAB; mouse; rat; blinded/not blinded assessment of the outcome #	Animal model (Q = 9.21; df = 3; *p* = 0.02); species (Q = 7.95; df = 1; *p* = 0.005); blinding (Q = 14.37; df = 1; *p* = 0.0001)

*—the significant components of individual protocols (animal model, species; drug; its dose or route of administration; early (preventive) or late (therapeutic regimen)) are demonstrated only; Q measure (*p* < 0.05)—indicates the significant heterogeneity between studies; **—imatinib given by inhalation or intratracheally displayed significant improvement in PH manifestation as compared to intragastric administration (Q = 29.45; df = 1; *p* < 0.0001); #—the analysis concerns assessments of RV hypertrophy and PA remodeling; BLEO—bleomycin; CH—chronic hypoxia; HEM—hemodynamic parameters featuring mean pulmonary artery pressure (mPAP), right ventricle systolic pressure (RVSP), or mean right ventricle pressure (RVP); LP—left pneumonectomy; MCT—monocrotaline; Nx—normoxia; PAB—pulmonary artery banding; RVH—right ventricle hypertrophy; remodel—pulmonary artery remodeling; SU—Sugen. ↓; (↑; ↔)—decrease (increase; no difference) in the parameter in the Intervention group as compared to placebo.

**Table 3:** An overview of efficacy of small-molecule protein kinase inhibitors in adult PAH in clinical trials.

Drug	Target	Type of Study	Population (% Female, Age)	Intervention and Comparator	Effects	Reference
Fasudil	ROCK	Prospective randomized controlled study	Adult patients with CHD-PAH; II, III, or IV FC-WHO (60%; mean age, 30 mg group: 36.6 ± 13.7 yrs.; 60 mg group: 38.9 ± 17.2 yrs.)	Fasudil: 30 or 60 mg once daily (iv)	↓mPAP; ↓PVR (60 mg); no SAEs	[115]
Fasudil	ROCK	Prospective, randomized, controlled, cross-over study	Adult patients with IPAH, PAH-CTD, or PAH due to repaired CHD; II or III FC-WHO (82%; mean age, 39 ± 13 yrs.)	Fasudil: 1 mg/min for 30 min (iv) vs. iloprost (5 ug, inh)	↓ mPAP and PVR in fasudil and iloprost group; ↑CO and oxygen saturation compared with iloprost	[114]
Fasudil	ROCK	A phase 2a randomized, double-blind, placebo-controlled study	Adult patients with PAH due to repaired CHD; PoPH; I, II, or III FC-WHO (69%; median age, placebo: 51.4 ± 16.2 yrs. and fasudil: 47.4 ± 14.2 yrs.)	Fasudil (orally): 2 to 6 capsules/day, once every 3 days vs. placebo; 12 weeks	↔6MWD; PVR; mPAP; CO and arterial oxygen saturation; SAEs: 0.9% (fasudil) and 0.16% (placebo)	[109]
Fasudil	ROCK	Prospective, open-label study	Adult patients with IPAH; PAH-CTD; PAH due to repaired CHD; PoPH; I, II, or III FC-WHO (86.7%; mean age, 45 (24−75 yrs.))	Fasudil: inhaled at 30 mg (10 min)	↓mPAP; PVR/SVR; ↔PVR	[108]
Imatinib	BCR-ABL	A phase 3, randomized, placebo-controlled, double-blind study	Adult patients with I/HPAH; PAH-CTD; PAH due to drugs and/or toxins/chemicals; PAH-HIV or PAH due to repaired CHD; II, III, or IV FC-WHO (18−75 yrs.)	Imatinib (orally): 200 mg once daily for two weeks, increased to 400 mg once daily, if well tolerated vs. placebo, for 24 weeks	↑RV function (TA S’; RV Tei index) and ↑LV size and LV early diastolic relaxation	[116]
Imatinib	BCR-ABL	A phase 2/3, randomized, placebo-controlled, double-blind, pilot study	Adult patients with I/HPAH; PAH-CTD; PAH due to repaired CHD; PAH due to diet or drugs; II, III, or IV FC-WHO (placebo: 89% and imatinib: 77%; mean age, placebo: 48.5 ± 13.0 yrs.; imatinib: 50.9 ± 13.7 yrs.)	Imatinib (orally): 200 mg once daily for two weeks, increased to 400 mg once daily, if well tolerated vs. placebo, for 24 weeks	↔6MWD; ↓PVR; ↑CO; SAEs: 39% (imatinib) and 23% (placebo)	[110]
Imatinib	BCR-ABL	A phase 3, randomized placebo-controlled, double-blind study (IMPRES)	Adult patients with IPAH/HPAH; PAH-CTD (excl. marked pulmonary fibrosis); II, III, or IV FC-WHO (placebo: 71%; imatinib: 64%; mean age, placebo: 44.2 ± 15.7 yrs.; imatinib: 44.4 ± 15.3 yrs.)	Imatinib (orally): 200 mg once daily for two weeks, increased to 400 mg once daily, if well tolerated vs. placebo, for 24 weeks	↑6MWD; ↓PVR; ↔functional class, time to clinical worsening, and mortality; SAEs: 44% (imatinib) and 30% (placebo); discontinuations: 33% (imatinib) and 18% (placebo)	[9]
Imatinib	BCR-ABL	Prospective, open, pilot study	Adult patients with I/HPAH; PAH-CTD; PAH due to repaired CHD; PAH due to diet or drugs; II, III, or IV FC-WHO (81%; median age, placebo: 47 (18–77 yrs.); imatinib: 50 (18–77 yrs.)	Imatinib (orally): 100 mg once daily for 24 weeks, increased to 200 mg once daily, if well tolerated, for 12 weeks	↔CI; mPAP; PVR; ↓PDGF-BB; ↔VEGF	[113]
Imatinib	BCR-ABL	A phase 3, randomized, double-blind, follow-up long-term extension of AV-101 study (IMPAHCT-FUL)	Adult patients with IPAH/HPAH, PAH-CTD; III FC-WHO (80%; mean age, 57.4 ±19.9 yrs.)	AV-101 (imatinib) administered via dry powder inhalation (at low, medium, and high dose)	Terminated	[121]
Imatinib	BCR-ABL	A phase 1/2 design comprising dose finding and single-arm efficacy	Adult patients with IPAH/HPAH, PAH-CTD; PAH due to drugs (both sexes; 18−80 yrs.)	Imatinib (orally): 100 mg, 200 mg, 300 mg, and 400 mg once daily for four weeks (part I); the best-tolerated dose administered for 24 weeks (part II)	Recruiting	[119]
Imatinib	BCR-ABL	Observational, prospective study	Adult patients with I/HPAH; PAH-CTD; PAH due to drugs and/or toxins/chemicals; PAH-HIV or PAH due to repaired CHD (both sexes; 18−80 yrs.)	Imatinib (orally): 400 mg once daily for 24 weeks	↓Functional class; mPAP; PVR; ↑CI; incidence of SDH was 2 per 37 patient-years	[117]
Imatinib via AV-101 dry powder inhalation	BCR-ABL	Phase 1, placebo-controlled, randomized, single (SAD) and multiple ascending dose (MAD)	Adult, healthy participants (53%, 18–59 yrs.)	Imatinib; SAD: 1 mg, 3 mg, 10 mg, 30 mg, 90 mg (inh); MAD: 10 mg, 30 mg, 90 mg (inh, twice daily, 7 days ) vs. imatinib (400 mg, orally) and placebo	Systemic exposure to imatinib delivered as an inhaled dry powder was significantly lower than with oral administration of imatinib (400 mg, per os). Drug was well-tolerated	[111]
Imatinib via AV-101 dry powder inhalation	BCR-ABL	A phase 2b/3, randomized, double-blind, placebo-controlled, dose-ranging, and confirmatory study	Adult, healthy participants (both sexes; 18−75 yrs.)	AV-101 (imatinib) administered via dry powder inhalation (at low, medium, and high dose (phase 2b) and at optimal dose selected in phase 2b (phase 3)) vs. placebo for 24 weeks	Completed, no results posted	[120]
nab-Sirolimus (nab-rapamycin, albumin-bound rapamycin ABI-009)	mTOR	A phase 1/1b, open-label study	Adult patients with I/HPAH; PAH-CTD; PAH due to drugs and/or toxins/chemicals; PoPH; I, II, III, or IV FC-WHO (69%; median age, 41 (34–52 yrs.))	nab-Sirolimus; 16 weeks	Not provided	[125]
Nilotinib	BCR-ABL	A phase 2, randomized, double-blind, placebo-controlled, efficacy, safety, tolerability, and PK study	Adult patients with IPAH; PAH-CTD; II, III, or IV FC-WHO (73.3%; 39−69 yrs.)	Nilotinib: oral administration of 50 mg (twice a day) for 2 weeks, 150 mg (twice a day) for 2 weeks followed by 300 mg (twice a day) for 24 weeks vs. placebo	Terminated due to SAEs	[122]
Seralutinib (dry powder inhalation)	PDGFR/c-Kit/BMPR2	A phase 3, randomized, double-blind, placebo-controlled study (PROSERA)	Adult patients with PAH; II or III FC-WHO (both sexes; 18 yrs. or older)	Seralutinib at 90 mg administered via generic dry powder inhaler for up to 48 weeks	Recruiting	[123]
Seralutinib (dry powder inhalation)	PDGFR/c-Kit/BMPR2	Open-label extension study that will evaluate the long-term effects of seralutinib	Adult patients with PAH who have completed GB002 PAH study (both sexes; 18−80 yrs.)	Capsule containing seralutinib inhaled twice a day for up to 144 weeks	Recruiting	[124]
Seralutinib (dry powder inhalation)	PDGFR/c-Kit/BMPR2	A phase 2, randomized, double-blind, placebo-controlled study (GB002 PAH study)	Adult patients with I/HPAH; PAH-CTD; PAH due to drugs and/or toxins/chemicals; or PAH due to repaired CHD; II or III FC-WHO (both sexes; 18−75 yrs.)	Seralutinib administered via generic dry powder inhaler for up to 24 weeks	↓ PVR; ↔6MWD; SAEs: 22.73% (seralutinib) and 14.29% (placebo)	[106,107]
Sorafenib	Raf-1, VEGF-R2, and PDGFR-β	A phase 1b, single-center, open-label dosing/cross-development study	Adult patients with I/HPAH; PAH-CTD; PAH due to drugs and/or toxins/chemicals or PAH due to repaired CHD; II or III FC-WHO (both sexes; 18−75 yrs.)	Sorafenib: 200 mg once daily orally, increased to 400 mg once daily, if well tolerated, for 16 weeks	↔6MWD, PVR; the most common AEs: moderate skin reactions and alopecia	[112]
Tacrolimus (FK506)	BMPR2	A phase 2, randomized, double-blind, placebo-controlled, safety and efficacy study	Adult patients with I/HPAH; PAH-CTD; PAH due to drugs and/or toxins/chemicals; I, II, or III FC-WHO (83%; 28−77 yrs.)	Tacrolimus: 3–5 ng/mL; 2–3 ng/mL; or <2.0 ng/mL blood level, for 18 weeks (1.5 mg by mouth once daily with dose adjustment)	FK506 was well tolerated, with nausea/diarrhea being the most commonly reported AEs	[118]

FC-WHO—World Health Organization (WHO) Functional Class; BMPR2—bone morphogenetic protein receptor 2; CHD—congenital heart disease; IPAH—idiopathic pulmonary arterial hypertension; HPAH—heritable pulmonary arterial hypertension; PAH-CTD—pulmonary arterial hypertension associated with connective tissue disease; PDGF-BB—platelet-derived growth factor type BB; PoPH—portopulmonary hypertension; PVR—pulmonary vascular resistance; SAE—serious adverse event; SDH—subdural hematoma; SVR—systemic vascular resistance; TGFBR—receptor for transforming growth factor beta; VEGF—vascular endothelial growth factor. ↓; (↑; ↔)—decrease (increase; no difference) in the parameter in the Intervention group as compared to placebo.

**Table 4:** Take-home messages.

Pulmonary vascular remodeling driven by abnormal vascular cell proliferation and apoptosis has been now recognized to play a critical role in progression of pulmonary arterial hypertension (PAH)
Protein kinases participate in the pathogenesis of a number of malignancies in addition to various autoimmune, inflammatory, nervous, and cardiovascular diseases
While recent clinical evaluations have yielded unsatisfactory results, some small-molecule protein kinase inhibitors have nevertheless been of great interest for studies on PAH
In preclinical studies, most of the reviewed molecules are able to control migration, proliferation, and survival in PASMCs and can target inflammatory, immune, and fibrotic pathways implicated in the pathology of PAH
Recent clinical studies have addressed molecules that affect multiple networks such as PDG-FRα/β/CSF1R/c-KIT/BMPR2 or FKBP12/mTOR in PAH
Researchers also focus on achieving satisfactory efficacy and safety profiles using innovative inhalation formulations (imatinib, seralutinib)
Different preclinical studies based on the same molecule can demonstrate relatively high heterogeneity and the following points are important: animal model that reflects human disease and impaired signaling pathways in relation to the molecule being tested, quality of reporting of experimental conditions, blinding the researcher, etc.
Standardized animal models can reduce inter-study heterogeneity and thereby facilitate successful identification of candidate drugs for further evaluations in PAH

## Data Availability

Authors can confirm that all relevant data are available in the article’s Supplementary Files and on request from the corresponding author.

## Supplementary Materials

The following supporting information can be downloaded at: https://www.mdpi.com/article/10.3390/ijms252312858/s1.
