# Supplementary material for: Searching for Old and New Small-Molecule Protein Kinase Inhibitors as Effective Treatments in Pulmonary Hypertension—A Systematic Review
Source: Int J Mol Sci. 2024 Nov 29;25(23):12858. doi: 10.3390/ijms252312858 (PMC11641621; doi:10.3390/ijms252312858)
Supplement: Supplementary file 1 [file ijms-25-12858-s001.zip › Supplementary Material (Search strategy+Data analysis).pdf]

Supplementary Material. Search strategy.

The databases: PUBMED, EBSCO(MEDLINE and website: clinical trials.gov;

Filters: from Jan 01 2001 Oct 31 2023

were searched with no language restrictions using the following search terms in titles and abstracts:

(PULMONARY HYPERTENSION **AND** SMALL MOLECULE PROTEIN KINASE INHIBITOR)

OR

(PULMONARY HYPERTENSION) **AND** (ABROCITINIB **OR** ACALABRUTINIB **OR** AFATINIB **OR** ALFLUTINIB **OR** ALECTINIB **OR** ALMONERTINIB **OR** APATINIB **OR** ASCIMINIB **OR** AVAPRITINIB **OR** AXITINIB **OR** BARICITINIB **OR** BELUMOSUDIL **OR** BINIMETINIB **OR** BOSUTINIB **OR** BRIGATINIB **OR** CABOZATINIB **OR** CAPMATINIB **OR** CATEQUENTINIB **OR** CERITINIB **OR** COBIMETINIB **OR** CRIZOTINIB **OR** DABRAFENIB **OR** DACOMITINIB **OR** DASATINIB **OR** DELGOCITINIB **OR** DOVITINIB **OR** ENCORAFENIB **OR** ENTRECTINIB **OR** ERDAFITINIB **OR** ERLOTINIB **OR** EVEROLIMUS **OR** FASUDIL **OR** HA-1007 **OR** FEDRATINIB **OR** FILGOTINIB **OR** FLUMATINIB **OR** FOSTAMATINIB **OR** FRUQUINTINIB **OR** FURMONERTINIB **OR** FUTIBATINIB **OR** GEFITINIB **OR** GILTERITINIB **OR** IBRUTINIB **OR** ICOTINIB **OR** IMATINIB **OR** STI571 **OR** INFIGRATINIB **OR** LAPATINIB **OR** LAROTRECTINIB **OR** LAZERTINIB **OR** LENVATINIB **OR** LORLATINIB **OR** MASITINIB **OR** MIDOSTAURIN **OR** MOBOCERTINIB **OR** NERATINIB **OR** NETARSUDIL **OR** NILOTINIB **OR** NINTEDANIB **OR** OLMUTINIB **OR** OSIMERTINIB **OR** PACRITINIB **OR** PALBOCICLIB **OR** PAZOPANIB **OR** PEFICITINIB **OR** PEMIGATINIB **OR** PEXIDARTINIB **OR** PONATINIB **OR** PRALSETINIB **OR** PYROTINIB **OR** ORELABRUTINIB **OR** RADOTINIB **OR** RAPAMYCIN **OR** REGORAFENIB **OR** RIBOCICLIB **OR** RIPASUDIL **OR** RIPRETINIB **OR** RIVOCERANIB **OR** RUXOLITINIB **OR** SAVOLITINIB **OR** SELPERCATINIB **OR** SELUMETINIB **OR OR** SERALUTINIB **OR** GB002 **OR** SIMOTINIB **OR** SIROLIMUS **OR** SORAFENIB **OR** SUNITINIB **OR** SURUFATINIB **OR** SURAMIN **OR** TACROLIMUS **OR** FK506 **OR** TEMSIROLIMUS **OR** TEPOTINIB **OR** TIRABRUTINIB **OR** TIVOZANIB **OR** TOCERANIB **OR** TOFACITINIB **OR** TRAMETINIB **OR** TRILACICLIB **OR** TUCATINIB **OR** UPADACITINIB **OR** VANDETANIB **OR** VEMURAFENIB **OR** Y27632 **OR** ZANUBRUTINIB)

OR

(PULMONARY HYPERTENSION) **AND** (PDGF **OR** PLATELET-DERIVED GROWTH FACTOR **OR** TGF **OR** TRANSFORMING GROWTH FACTOR **OR** EGF **OR** EPIDERMAL GROWTH FACTOR **OR** VEGF **OR** VASCULAR ENDOTHELIAL GROWTH FACTOR **OR** FGF **OR** FIBROBLAST GROWTH FACTOR **OR** SRC TYROSINE KINASE **OR** BCR-Abl **OR** mTOR **OR** ROCK **OR** Rho-kinase)

Supplementary Material. Data analysis

Data extraction, selection and analysis

The PUBMED, EBSCO (MEDLINE) and clinicaltrials.gov databases for were searched for preclinical (full-text paper) and clinical studies (abstract, full-text paper) regarding the ability of therapeutic agents targeting kinase families to reverse pulmonary hypertension. The search included preclinical studies addressing a wide spectrum of rodent models and experimental protocols on PH. They must have reported alterations in at least one PH-related parameter due to chronic exposure to an individual protein kinase inhibitor. All papers were published from January 2001 to October 2023.

Two independent reviewers (MJ-S, PG) searched the literature, extracted the data and performed quality assessment. This was done independently; any differences were resolved until consensus was reached.

The search (secondary aim) also included prospective clinical studies (recruiting, on-going, completed or terminated) including adult healthy participants or adult subjects with PAH, with primary outcomes defined as safety or efficacy (such as improvements in 6MWD, hemodynamic parameters or survival) of an individual small-molecule protein kinase inhibitor.

To allow further quantitative assessments, the following preclinical data were recorded, according to PICO, as described previously [1]. Other recorded data included PH-related lesions associated with hemodynamic parameters, such as mean pulmonary artery pressure – mPAP, systolic right ventricle pressure – RVSP, cardiac output – CO, cardiac index – CI, systemic blood pressure – BP. In addition, right ventricle hypertrophy (RVH), the degree of pulmonary artery (PA) remodeling, and the number of animals surviving the experiment were recorded [1]. The exclusion criteria for preclinical protocols were as follows: (a) review articles, (b) studies using in vitro, ex vivo models, only, (c) studies using animals other than rodents, (d) protocols intended to evoke cardiotoxicity or non-PH disease, (e) protocols that not reported PH-related parameters, (f) studies not addressing effects of therapeutic agents on PH, (g) studies reporting effects of gene therapy. The exclusion criteria concerned also clinical protocols where individual tyrosine kinase inhibitor was investigated in non-PH indication.

The electronic databases were searched by two independent reviewers (MJ-S, PG), who extracted the relevant descriptive and numerical data and assessed the risk of bias using the SYstematic Review Centre for Laboratory animal Experimentation (SYRCLE) tool [2]. Publication bias was assessed by performing an Egger's weighted regression test and the Duval and Tweedie 'trim and fill' procedure.

Sensitivity analysis – leave-one-out method – was used to assess differences between subgroups, and to verify whether the result in individual protocols might be robust.

Data analysis was performed as described previously [3]. The mean (+/– SD, or +/–SEM), and number of animals per group (n) were recorded. Where necessary, standard errors of the mean (SEM) were converted to standard deviations (SD). If the study protocol provided the range of subjects (e.g., 6–11), the lowest number was used, in situation where the number of animals at the end of the study was lacked, the initial number of subjects (at randomization) was used.

The study intervention characteristics was tabulated (MS Excel) and each protocol was assigned a unique number. This unique number was then a part of the ID assigned to each variable included into meta-analysis. The following data were extracted from the eligible studies and are listed in S3Table (preclinical studies) and S5Table (clinical reports).

The D and R measures indicated changes in the mean response (X) by the animal PH subject receiving therapeutic intervention (PH+TREATMENT, so-called "Treatment”), and placebo-controlled animals with PH (PH+PLACEBO, so-called “Placebo”).

$$D = X_{PH+TREATMENT} - X_{PH+PLACEBO} \quad \text{(equation S1)}$$

$$R = \frac{X_{PH+TREATMENT}}{X_{PH+PLACEBO}} \quad \text{(equation S2)}$$

For better visualization, the reversal ratio (R) of PH, calculated according to equation 2, was given in tree-plots or meta-regression curves. An R-value of 1 indicated no dissimilarities between these two groups, while values R << 1 designated a more pronounced reduction in a particular parameter featuring disease in the Treatment group as compared to Placebo group (PH prevention or reversal). The hypothesis about the influence of the study protocol-related variables on the outcome result was tested by performing subgroup analyses (animal model, rodent species, therapeutic agent/kinase family, dosage schedule, route of administration) and meta-regression (agent dose, administration period).

Subgroup analysis was used to identify the presence and extent of statistical heterogeneity; and the heterogeneity between animal subgroups was indicated by a statistically-significant Cochran’s Q-score (P<0.05). The results from subgroup analyses were only interpreted when comparisons between at least 3 interventions per group could be performed.

Statistical analyses were performed within STATISTICA 13.1 (random-effects model).

References

1. Jasińska-Stroschein, M. Training programs in preclinical studies. The example of pulmonary hypertension. Systematic review and meta-analysis. PloS One 2022, 17, e0276875. https://doi.org/10.1371/journal.pone.0276875.

2. Hooijmans, C.R.; Rovers, M.M.; de Vries, R.B.; Leenaars, M.; Ritskes-Hoitinga, M.; Langendam, M.W. SYRCLE's risk of bias tool for animal studies. BMC Med Res Methodol 2014, 14, 43. https://doi.org/10.1186/1471-2288-14-43.

3. Jasińska-Stroschein, M. Searching for Effective Treatments in HFpEF: Implications for Modeling the Disease in Rodents. Pharmaceuticals (Basel, Switzerland) 2023, 16, 1449. https://doi.org/10.3390/ph16101449.
