# Supplementary material for: Searching for Old and New Small-Molecule Protein Kinase Inhibitors as Effective Treatments in Pulmonary Hypertension—A Systematic Review
Source: Int J Mol Sci. 2024 Nov 29;25(23):12858. doi: 10.3390/ijms252312858 (PMC11641621; doi:10.3390/ijms252312858)
Supplement: Supplementary file 1 [file ijms-25-12858-s001.zip › Supplementary Material.pdf]

Supplementary Material.

Searching for old and new small-molecule protein kinase in-hibitors as effective treatments in pulmonary hypertension.

Magdalena Jasińska-Stroschein, and Paulina Glajzner

Supplementary Table S1. Leave-one-out sensitivity analysis – the summary.

Target	Drug	Parameter (effect size)	Effect size (95%CI) (all studies)#	Change in standard error (%)		Number of influential studies
ALK	(S)-Crizotinib; (R)-Crizotinib	mPAP (D, difference in means)	-0.45 (-14.80; 13.90); NS	-9.81%	32.81	0
BCR-Abl	Dasatinib; Imatinib		-8.59 (-11.40; -5.78); P<0.0001	-14.57%	14.09%	0
EGFR	Erlotinib; Gefitinib; Icotinib; Lapatinib; PKI166		-	-	-	-
mTOR	Sirolimus		-10.77 (-18.03; -3.51); P=0.0036	-9.61%	16.02%	0
ROCK	Fasudil hydrochloride; Fasudil dichloroacetate		-13.01 (-15.14; -10.89); P<0.0001	-7.44%	10.46%	0
TGF	TGFBRII-Fc		-	-	-	-
VEGFR	BIBF1000		-13.41 (-19.65; -7.17); P<0.0001	-1.96%	28.83%	0
Jak-STAT	Ruxolitinib		-	-	-	-
FGFR	BIBF1000; Dovitinib		-15.68 (-23.16; -8.19); P<0.0001	-60.71%	70.26%	0
ALK	(S)-Crizotinib; (R)-Crizotinib; IN-1233; K02288	RVSP (D, difference in means)	-4.14 (-13.04; 4.76); NS	-24.10%	15.72%	1
			-3.81 (-14.11; 6.49)*; NS	-22.55%	18.45%	0
BCR-Abl	Dasatinib; Imatinib; Ponatinib		-15.26 (-20.04; -10.48); P<0.0001	-8.04%	17.71%	0
EGFR	Erlotinib; Gefitinib; Icotinib; Lapatinib		-20.39 (-28.35; -12.44); P<0.0001	-3.55%	9.78%	0
mTOR	PP1; Sirolimus		-7.35 (-10.65; -4.05); P<0.0001	-28.35%	11.19%	0
ROCK	Fasudil hydrochloride; Fasudil dichloroacetate; Y27632		-12.90 (-15.32; -10.47); P<0.0001	-7.58%	11.16%	0
TGF	TGFBRII-Fc		-19.42 (-25.38; -13.45); P<0.0001	-10.71%	26.42%	0
VEGFR	BIBF1000; Cabozatinib; Regorafenib; Sunitinib; Toceranib		-17.50 (-27.87; -7.16); P=0.0009	-32.86%	9.84%	0
Jak-STAT	Ruxolitinib		-7.43 (-12.07; -2.78) P=0.0017	35.62%	64.04%	0
FGFR	BIBF1000; Dovitinib; Infigratinib; Nintedanib		-13.65 (-26.06; -1.23); P=0.031	3.39%	18.40%	1
			-3.81 (-14.11; 6.49)*; NS	-47.31%	30.46%	0
ALK	(S)-Crizotinib; (R)-Crizotinib; IN-1233; K02288	RVH (R, response ratio)	0.84 (0.73; 0.97); P=0.0201	-30.37%	16.40%	1
			0.81 (0.73; 0.90)*; P=0.0001	-7.73%	45.07%	0
BCR-Abl	Dasatinib; Imatinib		0.79 (0.74; 0.83); P<0.0001	-6.10%	6.96%	0
EGFR	Erlotinib; Gefitinib; Icotinib; Lapatinib		0.77 (0.71; 0.84); P<0.0001	-4.87%	14.80%	0
mTOR	Everolimus; PP1; Sirolimus; Temsirolimus		0.78 (0.73; 0.84); P<0.0001	-5.38%	8.04%	0
ROCK	Fasudil hydrochloride; Fasudil dichloroacetate; Y2763		0.71 (0.66; 0.76); P<0.0001	-11.71%	5.40%	0
TGF	TGFBRII-Fc		0.74 (0.67; 0.81); P<0.0001	-23.07%	21.06%	0
VEGFR	BIBF1000; Cabozatinib; Regorafenib; Sunitinib; Toceranib		0.75 (0.69; 0.81); P<0.0001	-9.38%	7.41%	0
Jak-STAT	Ruxolitinib; JSI-124		0.85 (0.70; 1.03); NS	-52.35%	63.21%	0

FGFR	BIBF1000; Dovitinib; Infigratinib; Nintedanib		0.81 (0.70; 0.94): P=0.0068	-19.51%	13.81%	0
ALK	(S)-Crizotinib; (R)-Crizotinib; IN-1233; K02288	remodel (R, response ratio)	0.82 (0.62; 1.09); NS	-15.69%	19.55%	0
BCR-Abl	Dasatinib; Imatinib; Nilotinib		0.71 (0.64; 0.77); P<0.0001	-11.01%	10.20%	0
EGFR	Erlotinib; Gefitinib; Icotinib; Lapatinib		0.66 (0.60; 0.72); P<0.0001	-4.99%	5.80%	0
mTOR	PP1; Sirolimus		0.74 (0.67; 0.82); P<0.0001	-3.21%	12.66%	0
ROCK	Fasudil hydrochloride; Fasudil dichloroacetate		0.50 (0.44; 0.57); P<0.0001	-6.49%	3.95%	0
TGF	TGFBRII-Fc		0.62 (0.55; 0.71); P<0.0001	-45.10%	40.11%	0
VEGFR	BIBF1000; Regorafenib; Sunitinib; Toceranib		0.76 (0.72; 0.80); P<0.0001	-2.78%	7.23%	0
Jak-STAT	Ruxolitinib		0.85 (0.66; 1.10); NS	-36.32%	21.54%	0
FGFR	BIBF1000; Dovitinib; Infigratinib; Nintedanib		0.50 (0.34; 0.72); P=0.0002	-31.95%	28.04%	0
ALK	(S)-Crizotinib; (R)-Crizotinib; IN-1233; K02288	BP (D, difference in means)	-	-	-	-
BCR-Abl	Imatinib		-9.29 (-15.17; -3.42); P<0.0001	-8.98%	19.36%	0
EGFR	Erlotinib; Gefitinib; Lapatinib		1.18 (-1.12; 3.48); NS	-28.00%	16.34%	0
mTOR	Everolimus; PP1; Sirolimus; Temsirolimus		-	-	-	-
ROCK	Fasudil hydrochloride; Fasudil dichloroacetate		-0.089 (1.68; -3.38); NS	-21.89%	22.26%	0
TGF	TGFBRII-Fc		-	-	-	-
VEGFR	BIBF1000; Regorafenib		9.07 (1.18; 16.97); P=0.024	-7.48%	16.84%	1
			6.20 (-1.10; 13.50)*; NS	-42.57%	20.71%	0
Jak-STAT	Ruxolitinib		-1.31 (-4.89; 2.27); NS	-1.29%	39.76%	0
FGFR	BIBF1000; Nintedanib		2.91 (-2.96; 8.77); NS	-5.32%	49.63%	0
ALK	(S)-Crizotinib; (R)-Crizotinib	CO (CI) (R, response ratio)	0.89 (0.66; 1.21); NS	-20.13%	21.95%	0
BCR-Abl	Dasatinib; Imatinib; Nilotinib		1.15 (0.99; 1.33); P=0.067	-16.26%	11.37%	1
			1.19 (1.057; 1.34)*; P=0.0042	-13.14%	15.48%	0
EGFR	Erlotinib; Gefitinib; Lapatinib		1.10 (1.05; 1.15); P<0.0001	5.16%	19.79%	0
mTOR	Everolimus; PP1; Sirolimus; Temsirolimus		-	-	-	-
ROCK	Fasudil hydrochloride; Fasudil dichloroacetate		-	-	-	-
TGF	TGFBRII-Fc		-	-	-	-
VEGFR	BIBF1000; Regorafenib; Sunitinib		1.31 (1.23; 1.41); P<0.0001	-7.06%	39.55%	0
Jak-STAT	Ruxolitinib		1.29 (1.10; 1.53); P=0.0023	-10.85%	78.61%	0
FGFR	BIBF1000; Nintedanib		1.15 (1.03; 1.28); P=0.011	-13.91%	100.71%	1
			1.06 (0.84; 1.33)*; NS	-17.01%	98.88%	0

* - after removing one study; # – at least 3 interventions must have been included into analysis

Supplementary Table S2. Results from publication bias funnel plot and 'trim and fill analysis'

Target	Drug	Parameter (effect size)	Egger regression P-value#	Imputed (trim and fill)
All	All	mPAP (D, difference in means)	P=0.002	3
		RVSP (D, difference in means)	P<0.0001	0
		RVH (R, response ratio)	P<0.0001	0
		remodel (R, response ratio)	P<0.0001	0
		BP (D, difference in means)	NS	1
		CO (CI) (R, response ratio)	NS	0
ALK	(S)-Crizotinib; (R)-Crizotinib	mPAP (D, difference in means)	NS	0
BCR-Abl	Dasatinib; Imatinib		NS	0
EGFR	Erlotinib; Gefitinib; Icotinib; Lapatinib; PKI166		-	-
mTOR	Sirolimus		NS	0
ROCK	Fasudil hydrochloride; Fasudil dichloroacetate		NS	0
TGF	TGFBRII-Fc		-	-
VEGFR	BIBF1000		NS	0
Jak-STAT	Ruxolitinib		-	-
FGFR	BIBF1000; Dovitinib		NS	0
ALK	(S)-Crizotinib; (R)-Crizotinib; IN-1233; K02288	RVSP (D, difference in means)	NS	0
BCR-Abl	Dasatinib; Imatinib; Ponatinib		P<0.0001	4
EGFR	Erlotinib; Gefitinib; Icotinib; Lapatinib		NS	0
mTOR	PP1; Sirolimus		NS	1
ROCK	Fasudil hydrochloride; Fasudil dichloroacetate; Y27632		P<0.0001	5
TGF	TGFBRII-Fc		NS	0
VEGFR	BIBF1000; Cabozatinib; Regorafenib; Sunitinib; Toceranib		NS	0
Jak-STAT	Ruxolitinib		NS	0
FGFR	BIBF1000; Dovitinib; Infigratinib; Nintedanib		NS	0
ALK	(S)-Crizotinib; (R)-Crizotinib; IN-1233; K02288	RVH (R, response ratio)	NS	0
BCR-Abl	Dasatinib; Imatinib		NS	0
EGFR	Erlotinib; Gefitinib; Icotinib; Lapatinib		NS	0
mTOR	Everolimus; PP1; Sirolimus; Temsirolimus		NS	0
ROCK	Fasudil hydrochloride; Fasudil dichloroacetate; Y2763		P<0.005	0
TGF	TGFBRII-Fc		NS	0
VEGFR	BIBF1000; Cabozatinib; Regorafenib; Sunitinib; Toceranib		NS	0

Jak-STAT	Ruxolitinib; JSI-124		NS	0
FGFR	BIBF1000; Dovitinib; Infigratinib; Nintedanib		NS	1
ALK	(S)-Crizotinib; (R)-Crizotinib; IN-1233; K02288	remodel (R, response ratio)	NS	0
BCR-Abl	Dasatinib; Imatinib; Nilotinib		P=0.009	0
EGFR	Erlotinib; Gefitinib; Icotinib; Lapatinib		P=0.03	0
mTOR	PP1; Sirolimus		NS	0
ROCK	Fasudil hydrochloride; Fasudil dichloroacetate		P<0.001	0
TGF	TGFBRII-Fc		NS	0
VEGFR	BIBF1000; Regorafenib; Sunitinib; Toceranib		P<0.0001	8
Jak-STAT	Ruxolitinib		NS	0
FGFR	BIBF1000; Dovitinib; Infigratinib; Nintedanib		NS	0
ALK	(S)-Crizotinib; (R)-Crizotinib; IN-1233; K02288	BP (D, difference in means)	-	-
BCR-Abl	Imatinib		NS	0
EGFR	Erlotinib; Gefitinib; Lapatinib		P=0.03	0
mTOR	Everolimus; PP1; Sirolimus; Temsirolimus		-	-
ROCK	Fasudil hydrochloride; Fasudil dichloroacetate		P=0.049	3
TGF	TGFBRII-Fc		-	-
VEGFR	BIBF1000; Regorafenib		NS	0
Jak-STAT	Ruxolitinib		NS	0
FGFR	BIBF1000; Nintedanib		NS	0
ALK	(S)-Crizotinib; (R)-Crizotinib	CO (CI) (R, response ratio)	NS	0
BCR-Abl	Dasatinib; Imatinib; Nilotinib		NS	1
EGFR	Erlotinib; Gefitinib; Lapatinib		P=0.04	0
mTOR	Everolimus; PP1; Sirolimus; Temsirolimus		-	-
ROCK	Fasudil hydrochloride; Fasudil dichloroacetate		-	-
TGF	TGFBRII-Fc		-	-
VEGFR	BIBF1000; Regorafenib; Sunitinib		NS	0
Jak-STAT	Ruxolitinib		NS	0
FGFR	BIBF1000; Nintedanib		NS	0

# – at least 3 interventions must have been included into analysis

Supplementary Table S3. An overview of preclinical studies – characteristics report.

1st Author (year)	Species	Race	Animal model	Intervention	Comparator	Protocol	Drug administration period (days)
Abe K (2004)	mouse	C57	CH, 10%O_2 (21 days)<br>eNOS-/- CH, 10%O_2 (21 days)	Fasudil hydrochloride	100 mg, per os	early	21
Abe K (2005)	rat	Sprague-Dawley	MCT (60 mg, ip)	Fasudil hydrochloride	30 mg and 100 mg, ig	early<br>late	21<br>42
Akagi S (2015)	rat	Sprague-Dawley	MCT (60 mg, ip)	Imatinib	1 mg, inh	early	21
Ambade AS (2019)	rat	Wistar	MCT (60 mg, ip)	BIBF1000	50 mg, per os	early<br>late	21
Atlı Ö (2017)	rat	Sprague-Dawley	MCT (60 mg, ip)	Everolimus	3 mg, ig	late	21
Awada C (2021)	rat	Sprague-Dawley	SU (20 mg)+CH (10%O_2, 21 days)<br>MCT (60 mg, ip)<br>MCT (60 mg, ip)	R-crizotinib	100 mg, ig	late<br>early<br>late	14<br>28<br>7
Baumgart B (2017)	rat	Sprague-Dawley	MCT (70 mg, ip)	Dasatinib<br>Imatinib	8 mg, per os<br>30 mg, per os	late	14
Bei Y (2013)	mouse	C57/BL6	bleomycin (3.3 U/kg, inh)	Fasudil hydrochloride	30 mg, ip	early	7, 14 21
Boehm M (2021)	mouse	C57Bl6/J	PAB	Tacrolimus (FK506)	0.05 mg, sc	late	35
Chen R (2023)	mouse	C57/BL6	SU (20 mg)+CH (10%O_2, 42 days)<br>CH (10%O_2, 28 days)	BI 6727	50 mg, iv	late	21<br>28
Cheng Y (2018)	rat	Sprague-Dawley	MCT (40 mg, ip)	Phps-1	1 mg, ip	late	14
Ciuclan L (2012)	mouse	C57/BL5	SU (20 mg)+CH (10%O_2, 21 days)<br>(+Tph1-/-)	Imatinib	200 mg, ig	early	21
Dahal BK (2010)	rat	Sprague-Dawley	MCT (60 mg, ip)<br>MCT (60 mg, ip)<br>MCT (60 mg, ip)	Gefitinib<br>Lapatinib<br>Erlotininb	10 and 30 mg, ig<br>5 and 10 mg, ig<br>5 and 10 mg, ig	late	14

			CH (10%O_2, 21 days)<br>CH (10%O_2, 21 days)<br>CH (10%O_2, 21 days)	Gefitinib<br>Lapatinib<br>Erlotininb	150 mg, ig<br>100 mg, ig<br>50 mg, ig		
Dai ZK (2011)	rat	Wistar	aorta banding	Fasudil hydrochloride	30 mg, sc	early<br>late	28<br>28
de Raaf MA (2016)	rat	Sprague-Dawley	PAB	BIBF1000	50 mg, ig	late	28
Elias-Al-Mamun M (2013)	rat	Sprague-Dawley	MCT (60 mg, ip)	Fasudil hydrochloride	30 mg, per os	early<br>late	21<br>54
Felix NS (2019)	rat	Wistar	MCT (60 mg, ip)	Infigratinib	5 mg, ig	late	14
Fujiwara T (2023)	mouse	C57BL/6J	SU (20 mg)+CH (10%O_2, 21 days)	Cabozantinib	20 and 150 mg, ip	early	21
Galkin A (2022)	rat	Sprague-Dawley	SU (20 mg)+CH (10%O_2, 21 days)<br>SU (20 mg)+CH (10%O_2, 21 days)+Nx (21%O_2, 21 days)<br>LP+MCT (60 mg, 7 days before)	Seralutinib<br>Seralutinib<br>Imatinib<br>Seralutinib	2.5 and 4.6 mg, inh<br>12.8 mg, inh,<br>15 mg, ig<br>2.5 mg, inh	early	21<br>14<br>14<br>10
Garat CV (2013)	rat	Wistar-Kyoto	CH (10%O_2, 21days)	Fasudil hydrochloride	10 mg, iv	early	21
Gosal K (2014)	rat	Sprague-Dawley	CH (13%O_2, 21days)	Y27632<br>Fasudil hydrochloride	200mM, inh<br>30 mg, ip	late	7
Guignabert C (2009)	mouse		Tie2 PPARγ−/−	Imatinib	50 mg, ip		14
Guignabert C (2016)	rat	Wistar	MCT (40 mg, ip) or CH (10%O_2, 21 days)	Imatinib or Dasatinib	20 mg, ig<br>10 mg, ig	early	7
Guilluy C (2005)	rat	Wistar	CH	Fasudil hydrochloride	30 mg, ig	early	14
Gupta N (2017)	rat	Sprague-Dawley	SU (20 mg)+CH (10%O_2, 21 days)+Nx (21%O_2, 21 days)	Fasudil hydrochloride	3 mg, inh (saline)	late	21
Hardie WD (2008)	mouse	FVB/NJ	CCSP+DOX (62.5 mg,)	Gefitinib	100 mg, per os	early	28
Houssaini A (2016)	mouse		CH (10%O_2, 21 days)<br>SM22-5HTT+	Sirolimus	2.5 and 5 mg, ig	late	21
Houssaini A (2013)	rat	Wistar	MCT (60 mg, ip)	Sirolimus<br>Imatinib	5 mg, ig<br>100 mg, ig	early, late<br>early, late	7 or 21

Huang H (2019)	mouse	C57BL/6J	CH (10%O_2, 28 days)	MIF098	40 mg, ip	early	28
Ivanovska J (2017)	rat	Sprague-Dawley	CH (13%O_2, 21days)	Temsirolimus	2.5 mg, ip	early	21
Izikki M (2013)	rat	Wistar	MCT (60 mg, ip)	Suramin	10 mg, iv	early<br>late	21<br>14
Jasińska-Stroschein M (2015)	rat	Wistar	MCT (60 mg, ip)	Imatinib	20 mg, ig	late	14
Jasińska-Stroschein M (2014)	rat	Wistar	MCT (60 mg, ip)	Imatinib<br>Fasudil hydrochloride	50 mg, ig<br>15 mg, ig	late	14
Kang Z (2016)	rat	Sprague-Dawley	bleomycin (5 U/kg, inh)	Ponatinib	1 mg, per os	early	17<br>35
Karpov AA (2022)	rat	Wistar	sodium alginate MSs	Ruxolitinib	0.86, 2.58 and 4.58 mg per os	late	28
Kimishima Y (2021)	mouse	C57BL/6J	CH (10%O_2, 14 days)<br>JAK2V617F+ CH (10%O_2, 14 days)	K02288	12 mg, ip	early	14
Klein M (2008)	rat	Sprague-Dawley	MCT (60 mg, ip)	imatinib<br>sorafenib	50 mg, ig<br>10 mg, ig	late	14
Kojonazarov B (2017)	mouse	C57/BL6	PAB<br>CH (10%O_2, 35 days)	PH797804	5 mg, ig	early	14<br>35
Kojonazarov B (2013)	rat	Sprague-Dawley	MCT (60 mg, ip)<br>MCT (60 mg, ip)<br>PAB<br>PAB	Sorafenib<br>Sunitinib<br>Sorafeninb<br>Sunitinib	5 and 10 mg, ig<br>1 and 10 mg, ig<br>10 mg, ig<br>10 mg, ig	late	14
Leong ZP (2019)	rat	Wistar-Imamichi	MCT (60 mg, ip)	Masitinib	5, 15, 50 mg per os	late	14
Leong ZP (2018)	rat	Wistar-Imamichi	MCT (60 mg, ip)	Imatinib<br>Sorafenib	5, 15, 50 mg per os<br>1, 3 and 10 mg, per os	late	14
Leong ZP (2018)	rat	Wistar-Imamichi	MCT (60 mg, ip)	Toceranib<br>Sorafenib	3 and 10 mg, ig<br>10 and 30 mg, ig	late	14
Li C (2018)	rat		CH (10%O_2, 21 days)	Fasudil hydrochloride	30 mg, ip	early	21
Li F (2006)	rat	Wistar	shunt	Fasudil hydrochloride	30 mg, ip		14

Liu M (2012)	rat	Sprague-Dawley	MCT (60 mg, ip)<br>CH (10%O_2, 35 days)	Fasudil hydrochloride	10, 30 and 75 mg, ig	late	14
Liu P(2021)	rat	Sprague-Dawley	SU (20 mg)+CH (10%O_2, 21 days)	Fasudil dichloroacetate	15, 45 and 135 mg, ig	early	21
Liu P (2019)	mouse	C57/BL6	SU (20 mg)+CH (10%O_2, 28 days)<br>CH (10%O_2, 28 days)	PP1<br>Berberine	4 mg, ip<br>100 mg, ig	early	28
Long L (2009)	rat	Sprague-Dawley	MCT (60 mg, ip)<br>CH (10%O_2, 21 days)	IN-1233	20 mg, ip	early, late<br>early	21
Ma X (2017)	rat	Sprague-Dawley	CA-JV shunt	Sirolimus	2 mg, ip	early	84
Maurer B (2012)	mouse	C57/BL6	Fra-2 tg	Nilotinib	75 mg, ig		56
McMurtry MS (2007)	rat	Sprague-Dawley	MCT (60 mg, ip)	Sirolimus	2.5 mg, ig	late	16
McNair BD (2021)	mouse	C57Bl6	hypobaric hypoxia	Sirolimus	14 ppm, per os	early	28
Medarametla V (2014)	rat	Sprague-Dawley	MCT (60 mg, ip)<br>LP+MCT (50mg, ip)	Imatinib	20 mg/ml, inh	late	14<br>9 and 14
Merklinger SL (2008)	rat	Sprague-Dawley	MCT (60 mg, ip)	PKI166	50 mg, per os	late	14
Milara J (2018)	rat	Wistar	bleomycin (3.75 U/kg, inh)	JSI-124	1 mg, iv	late	31
Moreno-Vinasco L (2008)	rat	Dahl salt-sensitive	CH (10%O_2, 28 days)<br>SU (20 mg)+CH (10%O_2, 28 days)	Sorafenib	2.5 mg, ig	early	28
Mouchaers KT (2010)	rat		MCT (40 mg, ip)	Fasudil hydrochloride	100 mg, ig	early	14
Nagaoka T (2006)	rat	FHR	CH	Fasudil hydrochloride	30 mg, per os	early	70
Nishimura T (2001)	rat	Sprague-Dawley	LP+MCT (60 mg, 7 days before)	Sirolimus	2.5 mg, ig	early<br>late	10<br>20
Norton CE (2020)	rat	Sprague-Dawley	CH (10%O_2, 28 days)	Gefitinib	30 mg, per os	early	28
Novoyatleva T (2021)	rat	Sprague-Dawley	MCT (60 mg, ip)<br>SU (20 mg)+CH (10%O_2, 21 days)+Nx (21%O_2, 21 days)	R428	100 mg, ig	late	14
Ormiston ML (2013)	rat	F344	MCT (70 mg, ip)	Imatinib	50 mg, ip	late	14

Paddenberg R (2007)	mouse	FVB	CH (10%O_2, 21 days)	Sirolimus	3 mg, ip	early	21
Pankey EA (2012)	rat	Sprague-Dawley	CH (10%O_2, 21 days)<br>MCT (50 mg, ip)	Imatinib<br>Imatinib	10 mg, iv<br>50 mg, ip	early	21
Peng H (2022)	rat	Sprague-Dawley	MCT (60 mg, ip)	Sirolimus	2 mg, ip	late	14
Peng LY (2020)	rat	Sprague-Dawley	MCT (60 mg, ip)	Icotinib	15, 30 and 60 mg, ig	early	28
Pullamsetti SS (2012)	rat	Sprague-Dawley	MCT (60 mg, ip)	Dasatinib<br>Nilotinib<br>Imatinib	5 and 15 mg, ig<br>30 mg, ig<br>100 mg, ig	late	14
Qi L (2018)	rat		MCT (60 mg, ip)	Fasudil hydrochloride<br>Fasudil dichloroacetate	37.5 mg, per os<br>43.3 mg, per os	late	28
Rashid J (2018)	rat	Sprague-Dawley	SU (20 mg)+CH (10%O_2, 21 days)+Nx (21%O_2, 21 days)	Fasudil hydrochloride	3 mg, inh (saline)	late	21
Richter MJ (2018)	rat	Wistar-Kyoto	SU (20 mg)+CH (10%O_2, 21 days)+Nx (21%O_2, 14 days)	Nintedanib	50 mg, ig	late	14
Rol N (2019)	rat	Sprague-Dawley	SU (25 mg)+CH (10%O_2, 28 days)+Nx (21%O_2, 28 days)	Nintedanib	50 mg, ig	late	21
Schermuly RT (2005)	rat<br>mouse	Sprague-Dawley<br>C57/BL6	MCT (60 mg, ip)<br>CH (10%O_2, 35 days)	Imatinib	1, 10 and 50 mg, ip<br>50 and 100 mg, ip	late	14
Shi Y (2021)	rat	Sprague-Dawley	MCT (50 mg, ip)<br>SU (20 mg)+CH (10%O_2, 21 days)+Nx (21%O_2, 14 days)<br>MCT (60 mg, ip)<br>SU (20 mg)+CH (10%O_2, 21 days)+Nx (21%O_2, 14 days)	Imatinib<br>Imatinib<br>Sirolimus<br>Sirolimus	2 and 10 mg, ip	late	14
Spiekerkoetter E (2013)	mouse<br>rat	C57/BL6<br>Sprague-Dawley	CH (10%O_2, 21 days) +EC-Bmpr-/+<br>CH (10%O_2, 21 days)<br>SU (20 mg)+CH (10%O_2, 21 days)+Nx (21%O_2, 56 days)<br>MCT (60 mg, ip)	Tacrolimus (FK506)	0.05 mg, sc	early<br>late	21<br>21<br>42<br>42
Sun XZ (2015)	rat	Sprague-Dawley	CH (10%O_2, 21 days)	Fasudil hydrochloride	30 mg, ip	early	21

Tawara S (2007)	rat	Sprague-Dawley	MCT (60 mg, ip)	Fasudil hydrochloride	30 mg, ip	early	21
Tsutsumi T (2019)	rat	Sprague-Dawley	SU (20 mg)+CH (10%O_2, 21 days)+Nx (21%O_2, 14 days)	Nintedanib	50 mg, ig	late	14
Tu L (2012)	rat	Wistar	MCT (60 mg, ip)	Imatinib<br>Gefitinib<br>Dovitinib	50 mg, ip<br>50 mg, ip<br>30 mg, ip	late	14
Veeroju S (2021)	rat<br>mouse	Sprague-Dawley<br>C57BL/6J	MCT (60 mg, ip)<br>CH (10%O_2, 21 days)	Regorafenib	15 mg, ig<br>30 mg, ig	late	14
Vitry G (2021)	rat	Sprague-Dawley	MCT (60 mg, ip)<br>SU (20 mg)+CH (10%O_2, 21 days)+Nx (21%O_2, 14 days)	(S)-Crizotinib	100 nmol, inh	late	14
Wang AP (2015)	rat	Sprague-Dawley	CH (10%O_2, 21 days)	Sirolimus	1 mg, ig	early	21
Wang R (2022)	rat	Sprague-Dawley	CH (10%O_2, 21 days)	Dasatinib	10 mg, ip	early	28
Wang YX (2014)	rat	Sprague-Dawley	CH (10%O_2, 42 days)	Fasudil hydrochloride	10 and 30 mg, ig	early	14
Weiss A (2019)	rat	Sprague-Dawley	MCT (60 mg, ip)<br>SU (20 mg)+CH (10%O_2, 21 days)+Nx (21%O_2, 14 days)	Palbociclib	75 mg, ig	late	14
West JD (2016)	mouse		BMPR2 mutant	SB204741	1 mg, sc		28
Yamamura A (2019)	rat	Sprague-Dawley	MCT (60 mg, ip)	Imatinib<br>NPS2143	10 mg, ip<br>4.5 mg, ip	late	7
Yamamura A (2021)	rat	Sprague-Dawley	MCT (60 mg, ip)	KD02	1 mg, ip	late	14
Yasuda T (2010)	mouse	FVB/N	SM22-tet-BMPR2R899X	Fasudil hydrochloride	100 mg, per os		14<br>28
Yerabolu D (2021)	Rat<br>mouse	Sprague-Dawley<br>C57BL/6J	MCT (60 mg, ip)<br>CH (10%O_2, 21 days)	Ruxolitinib	30 and 90 mg, ig<br>60 and 180 mg, ig	late	14
Yu M (2022)	rat	Sprague-Dawley	MCT (60 mg, ip)	BCG-3111	40, 80, 160 mg, ig	early	21
Yung LM (2016)	rat	Sprague-Dawley	MCT 40 mg, ip)<br>CH (10%O_2, 21 days)<br>SU (20 mg)+CH (10%O_2, 21 days)+Nx (21%O_2, 21 days)	TGFBRII-Fc	5 mg, ip (twice a week)	early or late<br>early<br>late	18 or 21<br>21<br>21

	mouse	C57	CH (10%O_2, 21 days)			early	21
Zhou H (2006)	rat	Sprague-Dawley	MCT (100 mg, ip)	Sirolimus	2 mg, ig	early	28
Zhuang R (2018)	rat	Sprague-Dawley	ascending aorta banding	Fasudil hydrochloride	30 mg, ip	late	28

Supplementary Table S4. The influence of small molecule protein kinase inhibitors on the changes in PH-linked parameters

Drug	Effect# (stand. error)
BCG-3111	↓RVSP (D=-9.33; 0.67; P<0.0001)<br>↓RVH (R=0.70; 0.037; P<0.0001)<br>↓remodel (R=0.81; 0.027; P<0.0001)
BI 6727	↓RVSP (D=-10.0; 0.63; P<0.0001)<br>↓RVH (R=0.75; 0.035; P<0.0001)<br>↑CO (CI) (R=1.15; 0.052; P=0.0022)<br>↔BP (D=-1.0; 0.81; NS)
BIBF1000	↓RVSP (D=-23.48; 6.53; P=0.0003)<br>↓mPAP (D=-19.5; 1.50; P<0.0001)<br>↓RVH (R=0.77; 0.064; P=0.0014)<br>↑CO (CI) (R=1.23; 0.054; P<0.0001)<br>↑BP (D=8.5; 2.08; P<0.0001)
Cabozantinib	↔RVH (R=1.06; 0.046; NS)
(S)-Crizotinib	↓mPAP (D=-20.56; 3.98; P<0.0001)<br>↓RVSP (D=-20.0; 1.94; P<0.0001)<br>↔RVH (R=0.76; 0.17; NS)<br>↑CO (CI) (R=1.21; 0.058; P=0.0001)
(R)-Crizotinib	↑RVSP (D=15.73; 3.04; P<0.0001)<br>↑mPAP (D=13.08; 0.92; P<0.0001)<br>↔remodel (R=1.26; 0.24; NS)<br>↓CO (CI) (R=0.73; 0.11; P=0.039)
Dasatinib	↓RVSP (D=-20.96; 7.69; P=0.0064)<br>↔mPAP (D=-2.39; 4.47; NS)<br>↔RVH (R=0.87; 0.12; NS)<br>↔remodel (R=0.72; 0.17; NS)<br>↔CO (CI) (R=1.15; 0.15; NS)
Dovitinib	↓mPAP (D=-8.0; 1.0; P<0.0001)<br>↓RVH (R=0.67; 0.026; P<0.0001)
Erlotinib	↓RVSP (D=-19.31; 9.22; P=0.036)<br>↓RVH (R=0.80; 0.077; P=0.019)<br>↓remodel (R=0.82; 0.049; P=0.0008)<br>↔CO (CI) (R=1.06; 0.039; NS)<br>↓BP (D=-4.20; 2.17; P=0.053)
Everolimus	↓RVSP (D=-5.0; 1.0; P<0.0001)
Fasudil dichloroacetat e	↓mPAP (D= -10.33; 1.19; P<0.0001)<br>↓RVH (R=0.70; 0.054; P<0.0001)<br>↓remodel (R=0.45; 0.056; P<0.0001)<br>↔BP (D=-5.0; 8.67; NS)
Fasudil hydrochloride	↓mPAP (D= -13.26; 1.20; P<0.0001)<br>↓RVH (R=0.71; 0.025; P<0.0001)<br>↓remodel (R=0.58; 0.034; P<0.0001)<br>↔BP (D=0.05; 1.73; NS)
Gefitinib	↓RVSP (D=-21.96; 8.10; P=0.0067)<br>↓mPAP (D=-12.0; 1.0; P<0.0001)<br>↓RVH (R=0.71; 0.052; P<0.0001)<br>↓remodel (R=0.78; 0.045; P<0.0001)<br>↑CO (CI) (R=1.15; 0.048; P=0.0007)<br>↔BP (D=0.29; 1.27; NS)
Icotinib	↓RVSP (D= -14.67; 1.42; P<0.0001)<br>↓RVH (R=0.82; 0.069; P=0.02)<br>↓remodel (R=0.67; 0,089; P=0.0028)
Imatinib	↓RVSP (D=-14.64; 2.65; P<0.0001)[a]<br>↓mPAP (D=-10.44; 1.29; P<0.0001)[a]<br>↓RVH (R=0.77; 0.022; P<0.0001)[b]<br>↓remodel (R=0.87; 0.026; P<0.0001)<br>↓BP (D=-9.29; 3.0; P=0.002)<br>↔CO (CI) (R=1.16; 0.12; NS)
IN-1233	↓RVSP (D=-12,14; 3.95; P=0.0021)<br>↔RVH (R=0.84; 0.097; NS)
Infigratinib	↑RVSP (D=5.0; 2.5; P=0.045)<br>↓RVH (R=0.78; 0.045; P<0.0001)
JSI-124	↓RVH (R=0.59; 0.015; P<0.0001)
K02288	↓RVSP (D=-5.53; 1.50; P=0.0002)<br>↔RVH (R=0.93; 0.037; NS)<br>↔remodel (R=0.79; 0.15; NS)
KD02	↓RVSP (D=-7.0; 1.6; P<0.0001)
Lapatinib	↓RVSP (D=-17.66; 8.29; P<0.0001)<br>↓RVH (R=0.85; 0.052; P=0.0089)<br>↓remodel (R=0.84; 0.031; P<0.0001)<br>↑CO (CI) (R=1.10; 0.04; P=0.0125)<br>↔BP (D=2.46; 2.89; NS)
Masitinib	↓RVH (R=0.81; 0.037; P<0.0001)<br>↓remodel (R=0.67; 0.052; P<0.0001)
MIF098	↓RVSP (D=-17.5; 0.71; P<0.0001)<br>↓RVH (R=0.9; 0.040; P=0.017)
Nilotinib	↓RVSP (D=-22.0; 1.93: P<0.0001)<br>↓RVH (R=0.79; 0.023; P<0.0001)<br>CO (CI) (R=1.0; 0.03; NS)<br>↓remodel (R=0.71; 0.10; P=0.019)
Nintedanib	↔RVSP (D=-9.64; 6.98; NS)<br>↔RVH (R=0.93; 0.10; NS)<br>↔CO (CI) (R=1.03; 0.04; NS)<br>↔BP (D=-5.0; 3.16; NS)
Palbociclib	↓RVSP (D=-13.0; 1.54; P<0.0001)<br>↓RVH (R=0.68; 0.012; P<0.0001)<br>↓remodel (R=0.78; 0.022; P<0.0001)<br>↔BP (D=-5.6; 4.5; NS)
PH797804	↔RVSP (D=-2.06; 1.43; NS)
PK10453	↓RVSP (D= -28.99; 6.15; P<0.0001)<br>↓RVH (R=0.8; 0.011; P<0.0001)<br>↑CO (CI) (R= 2.69; 0.28; P<0.0001)
PKI166	↓mPAP (D=-19;2.03; P<0.0001)<br>↓RVH (R=0.83; 0.014; P<0.0001)<br>↑CO (CI) (R=2.69; 0.28; P<0.0001)
Ponatinib	↓RVSP (D=-6.19; 1.66; P=0.0002)<br>↓RVH (R=0.82; 0.038; P<0.0001)
PP1	↓RVSP (D=-10.0; 1.82; P<0.0001)<br>↓RVH (R=0.43; 0.022; P<0.0001)
R428	↔RVSP (D=13.5; 11.5; NS)<br>↓CO (CI) (R=0.60; 0.012; P<0.0001)
Regorafenib	↔RVSP (D=-11.48; 6.50; NS)<br>↓RVH (R=0.82; 0.027; P<0.0001)<br>↔CO (CI) (R=1.37; 0.24; NS)<br>↔BP (D=-0.5; 1.24; NS)
Ruxolitinib	↓RVSP (D=-7.43; 2.37; P=0.0017)

	↔RVH (R=0.94; 0.04; NS)<br>↔remodel (R=0.93; 0.070; NS)<br>↑CO (CI) (R=1.29; 0.11; P=0.0023)<br>↔BP (D=-0.77; 1.60; NS)
SB204741	↓RVSP (D=-10.0; 1.58; P<0.0001)
Seralutinib	↓RVSP (D=-27.08; 5.31; P<0.0001)<br>↓mPAP (D= -18.0; 1.89; P<0.0001)<br>↓RVH (R=0.67; 0.039; P<0.0001)<br>↑CO (CI) (R=1.31; 0.07; P<0.0001)
Sirolimus	↓RVSP (D=-7.70; 2.09; P=0.0002)<br>↓mPAP (D=-10.78; 3.70; P<0.0001)<br>↓RVH (R=0.80; 0.03; P<0.0001)<br>↓remodel (0.86; 0.066; P=0.056)<br>↑CO (CI) (R=1.09; 0.04; P=0.018)<br>↔BP (D=-1.0; 0.81; NS)
Sorafenib	↓RVSP (D=-20,89; 10.45; P=0.046)<br>↓mPAP (D= -9.88; 4.99; P=0.048)<br>↓RVH (R=0.65; 0.040; P<0.0001)
	↓remodel (R=0.86; 0.011; P<0.0001)<br>↑CO (CI) (R=1.33; 0.16; P=0.017)<br>↑BP (D=16.58; 6.76; P=0.014)
Sunitinib	↓RVSP (D=-20.30; 6.5; P=0.0018)<br>↓RVH (R=0.83; 0.039; P=0.0001)<br>↓remodel (R=0.95; 0.0049; P<0.0001)<br>↑CO (CI) (R=1.28; 0.16; P=0.04)
Suramin	↓mPAP (D=-20.0; 2.0; P<0.0001)<br>↓RVH (R=0.63; 0.033; P<0.0001)
Tacrolimus (FK506)	↓RVSP (D=-10.04; 3.73; P=0.0072)
TGFBRII-Fc	↓RVSP (D=-12.27; 3.21; P=0.0001)<br>↓RVH (R=0.79; 0.02; P<0.0001)<br>↓remodel (R=0.74; 0.02; P<0.0001)
Toceranib	↔RVSP (D=-0.91; 3.067; NS)<br>↓RVH (R=0.76; 0.071; P=0.0034)<br>↔remodel (R=0.89; 0.013; P<0.0001)
Y27632	↓RVSP (D=-12.0; 1.15; P<0.0001)

# – effect size expressed as D = difference in means or R = response ratio, where D > 0 or R>1 indicate increase in the value of the individual parameter as compared to animals with pulmonary hypertension receiving placebo; [a] – P<0.0001 for comparison between imatinib given per os and in inhalation (Q=16.68; df=1); [b] – P<0.0001 for comparison between imatinib given per os and in inhalation (Q=26.34; df=1)

Supplementary Table S5. An overview of clinical trials on small molecule protein kinase inhibitors in adult PAH – characteristics report.

1st Author (year)/NCT No	Type of study	Population (% female, age)	N	Intervention	Comparator	Outcomes
Frantz RP (2021)	A phase 2, randomized, double-blind, placebo-controlled study (GB002 PAH study)	Adult patients with I/HPAH; PAH-CTD; PAH due to drugs and/or toxins/chemicals; or PAH due to repaired CHD; II, or III FC-WHO (both sexes; 18–75 yrs.)	Seralutinib: n=44; placebo: n=42	Seralutinib inhaled orally twice a day for 24 weeks	placebo	Primary: change from baseline to 24 week in PVR; secondary: change from baseline to 24 week in 6MWD; safety and tolerability
Fujita H (2010)	Prospective, open label study	Adult patients with IPAH; PAH-CTD; PAH due to repaired CHD; PoPH; I, II or III FC-WHO (86.7%; mean age, 45 (24–75 yrs.))	Fasudil: n=15	Fasudil: inhaled at 30 mg (10 min)	-	Hemodynamic parameters at baseline and after 30 minutes of fasudil through right cardiac catheterization. Safety and tolerability.
Fukumoto Y (2013)	A phase IIa randomized, double-blind, placebo-controlled study	Adult patients with IPAH; PAH-CTD; PAH due to repaired CHD; PoPH; I, II or III FC-WHO (69%; median age, placebo 51.4±16.2 and fasudil: 47.4±14.2 yrs.)	Fasudil: n=11	Fasudil: 2 to 6 capsules/day, once every 3 days	Placebo	Change in hemodynamic parameters (mPAP, PVR, CO) and 6MWD from baseline after 12 weeks
Ghofrani HA (2010)	A phase 2/3, randomized, placebo-controlled, double-blind, pilot study	Adult patients with I/HPAH; PAH-CTD; PAH due to repaired CHD; PAH due to diet or drugs; II, III or IV FC-WHO (placebo: 89% and imatinib: 77%; mean age, placebo: 48.5 ± 13.0; imatinib: 50.9 ± 13.7 yrs.)	Imatinib: n=28; placebo: n=31	Imatinib: 200 mg once daily for two weeks, increased to 400 mg once daily, if well tolerated; 24 weeks	placebo	Primary: efficacy (change from baseline to 24 week in 6MWD); safety and tolerability; secondary: hemodynamics and functional class
Gillies H (2023)	Phase 1, placebo-controlled, randomised single (SAD) and multiple ascending dose (MAD)	Adult, healthy participants (53%, 18–59 yrs.)	SAD: n=48; MAD: n=34	Imatinib SAD: 1 mg, 3 mg, 10 mg, 30 mg, 90 mg (inh); MAD: 10 mg, 30 mg, 90 mg (inh, twice daily, 7 days)	placebo	Pharmacokinetics, safety and tolerability

Gomberg-Maitland M (2010)	A phase Ib, single-center, open-label dosing/cross-development study	Adult patients with I/HPAH; PAH-CTD; PAH due to drugs and/or toxins/chemicals or PAH due to repaired CHD; II, or III FC-WHO (both sexes; 18–75 yrs.)	Sorafenib: n=22	Sorafenib: 200 mg once daily, increased to 400 mg once daily, if well tolerated, for 16 weeks	-	Safety and exploratory assessment for preliminary evidence of therapeutic activity
Hatano M (2010)	Prospective, open, pilot study	Adult patients with I/HPAH; PAH-CTD; PAH due to repaired CHD; PAH due to diet or drugs; II, III or IV FC-WHO (81%; median age, placebo: 47 (18–77); imatinib: 50 (18–77) yrs.)	Imatinib: n=5	Imatinib: 100 mg once daily for 24 weeks, increased to 200 mg once daily, if well tolerated, for 12 weeks	-	Hemodynamics, exercise capacity, plasma PDGF-BB and VEGF
Hoeper MM (2013)	A phase 3, randomized placebo-controlled, double-blind study (IMPRES)	Adult patients with IPAH/HPAH; PAH-CTD (excl. marked pulmonary fibrosis); II, III or IV FC-WHO (placebo: 71%; imatinib: 64%; mean age, placebo: 44.2±15.7; imatinib: 44.4±15.3 yrs.)	Imatinib: n=103; placebo: n=99	Imatinib: 200 mg once daily for two weeks, increased to 400 mg once daily, if well tolerated; 24 weeks	placebo	Primary: efficacy (change from baseline to 24 week in 6MWD); safety and tolerability; secondary: hemodynamics, functional class, serum levels of NT-BNP, and time to clinical worsening
Jiang X (2014)	Randomized, controlled, crossover study.	Adult patients with IPAH; PAH-CTD or PAH due to repaired CHD; II or III FC-WHO (82%; mean age, 39±13 yrs.)	Fasudil: n=50	Fasudil: 1 mg/min for 30 min (iv)	Iloprost (5ug, inh)	Hemodynamic parameters at baseline and after 30 minutes of fasudil through right cardiac catheterization. Safety and tolerability.
NCT01179737	A phase 2, randomized, double-blind, placebo-controlled, Efficacy, Safety, Tolerability and PK study	Adult patients with IPAH; PAH-CTD; II, III or IV FC-WHO (73.3%; 39–69 yrs.)	Nilotinib: n=23	Nilotinib: 50 mg (twice a day) during 2 weeks, 150 mg (twice a day) during 2 weeks followed by 300 mg (twice a day) during 24 weeks	placebo	Efficacy, safety and pharmacokinetics
NCT02587325	A Phase 1/1b, open label study	Adult patients with I/HPAH; PAH-CTD; PAH due to drugs and/or toxins/chemicals; PoPH;	nab-sirolimus: n=15	Not provided	-	Primary: maximum-tolerated dose (MTD); dose-limiting toxicities (DLT)

		I, II, III or IV FC-WHO (69%; median age, 41 (34–52 yrs.))				
NCT04816604	A Phase 2, open-label extension study will evaluate the long-term effects of seralutinib	Adult patients with PAH who have completed GB002 PAH study (both sexes; 18–80 yrs.)		Seralutinib administered via generic dry powder inhaler up to 144 weeks	-	Number of participants with treatment emergent AEs
NCT05036135	A phase 2b/3, randomized, Double-Blind, Placebo-Controlled, 24-Week Dose Ranging and Confirmatory Study (IMPAHCT)	Adult, healthy participants (both sexes; 18–75 yrs.)	-	AV-101 (imatinib) administered via dry powder inhalation (at low, medium and high dose (phase 2b) and at optimal dose selected in phase 2b (phase 3)); 24 weeks	placebo	Change from baseline to 24 week in PVR (phase 2b); change from baseline to 24 week in 6MWD (phase 3); safety and tolerability
NCT05557942	A phase 3, randomized, double-blind, follow up long term extension of AV-101 Study (IMPAHCT-FUL)	Adult patients with IPAH/HPAH, PAH-CTD; III FC-WHO (80%; mean age, 57.4 ±19.9 yrs.)	-	AV-101 (imatinib) administered *via* dry powder inhalation (at low, medium and high dose	-	Long-term safety
NCT05934526	A Phase 3, randomized, double-blind, placebo-controlled Study (PROSERA)	Adult patients with PAH; II or III FC-WHO (both sexes; 18 yrs. or older)	-	Seralutinib administered via generic dry powder inhaler up to 48 weeks	placebo	Efficacy: change from baseline to 48 week in 6MWD; safety and tolerability
Ruan H (2019)	Prospective randomized controlled study.	Adult patients with CHD-PAH; II, III or IV FC-WHO (60%; mean age, 30 mg group: 36.6±13.7; 60 mg group: 38.9±17.2 yrs.)	Fasudil: n=60	Fasudil: 30 or 60 mg one daily	-	Hemodynamic parameters at baseline and after 30 minutes of fasudil through right cardiac catheterization. Safety and tolerability.
Shah AM (2015)	A phase 3, randomized placebo-controlled, double-blind study	Adult patients with I/HPAH; PAH-CTD; PAH due to drugs and/or toxins/chemicals; PAH-HIV or PAH due to repaired CHD; II, III or IV FC-WHO (18–75 yrs.)	Imatinib: n=103; placebo: n=99	Imatinib: 200 mg once daily for two weeks, increased to 400 mg once daily, if well tolerated; 24 weeks	placebo	RV function assessed by echocardiography.

Speich R (2015)	Observational, prospective study	Adult patients with I/HPAH; PAH-CTD; PAH due to drugs and/or toxins/chemicals; PAH-HIV; or PAH due to repaired CHD (both sexes; 18–80 yrs.)	Imatinib: n=15	Imatinib: 400 mg once daily for 24 weeks	-	Efficacy and safety
Spiekerkoetter E (2017)	A phase 2, randomized, double-blind, placebo-controlled, safety and efficacy study	Adult patients with I/HPAH; PAH-CTD; PAH due to drugs and/or toxins/chemicals; I, II or III FC-WHO (83%; 28–77 yrs.)	Tacrolimus: n=30	Tacrolimus: 3-5 ng/ml; 2-3 ng/ml; or < 2.0 ng/ml blood level, for 18 weeks	placebo	Primary: safety
Wilkins MR (2021)	A phase 1/2 design comprising dose finding and single-arm efficacy	Adult patients with IPAH/HPAH, PAH-CTD; PAH due to drugs (both sexes; 18–80 yrs.)	-	Imatinib: 100 mg, 200 mg, 300 mg and 400 mg once daily for four weeks (part I); the best tolerated dose administered for 24 weeks (part II)	-	Primary: identification of the best tolerated dose of imatinib (part I); the safety and efficacy of the best tolerated dose (PVR) (part II)

6MWD – 6-minute walking distance; CHD – Congenital heart disease; HPAH – Heritable pulmonary arterial hypertension; IPAH – Idiopathic pulmonary arterial hypertension; NT-BNP – N-terminal brain natriuretic peptide; PAH-CTD – Pulmonary arterial hypertension associated with connective tissue disease; PDGF-BB – Platelet derived growth factor type BB; PVR – Pulmonary vascular resistance; RV – right ventricular; VEGF – vascular endothelial growth factor

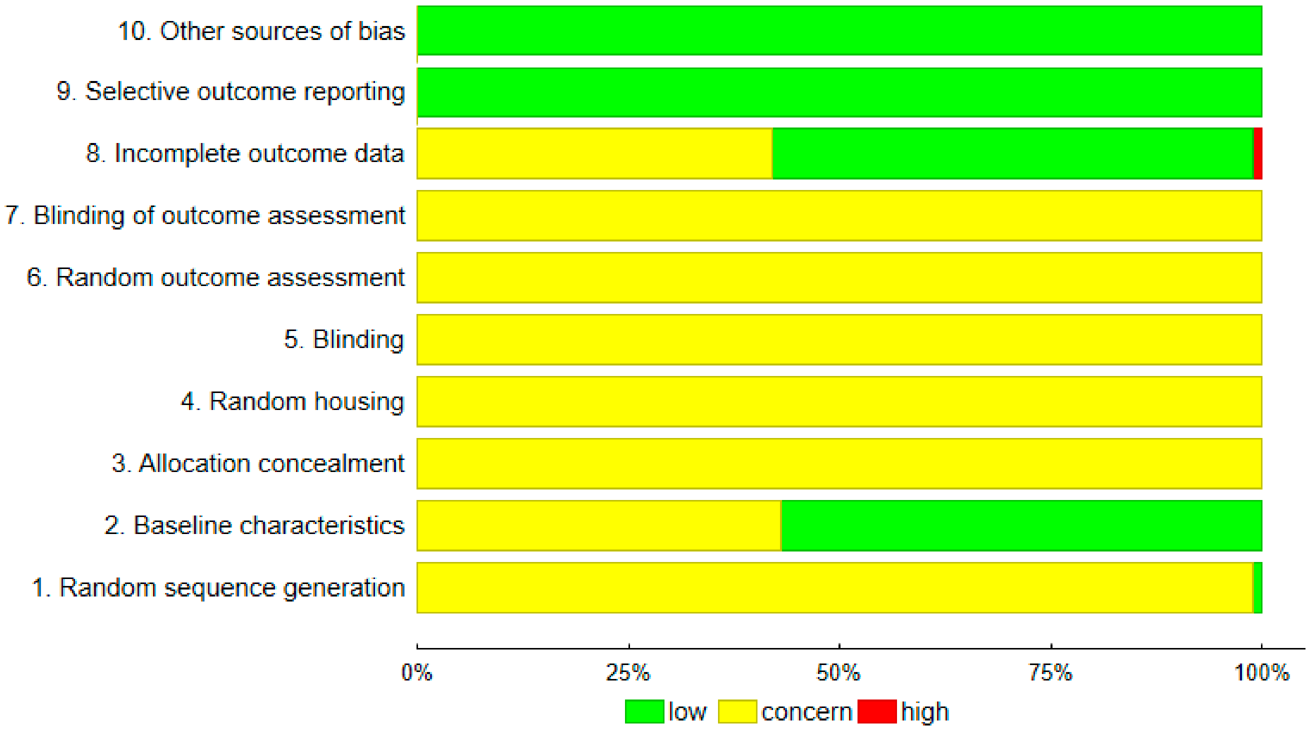

Supplementary Figure S1. Assessment of risk of bias.

Results of the risk of bias assessment (N=93 studies) according to the SYRCLE Risk of Bias strategy. In 54.8 percent of the experimental protocols, the animals were randomly allocated to the respective groups; however, no methods were specified (unclear risk of bias; concern). In the remaining studies, it was not reported whether the randomization process was carried out (unclear risk of bias; concnern). In 33.3 percent of papers, it was stated that the outcome assessment was blinded. This concerned mainly histomorphometric analyses. The studies did not provide any detailed information about the procedure used to blind researchers (unclear risk of bias; concnern). Fifty one of the 93 papers (54.8%) gave the baseline characteristics of the animal subjects – including complete information about animal age, sex, strain and genetic backgrorund (if needed) (low risk of bias). Also, 53 of the 93 experimental protocols (57.0%) reported the number of animals per each experimental group (before and at the end of the study) (low risk of bias).

A

Drug	Risk ratio (95%CI)	Weight %	N1 (Treatment)	N2 (Placebo)
BIBF1000	0.51 (0.38;0.68); P<0.0001	3.71%	48	46
Seralutinib	0.62 (0.55;0.71); P<0.0001	5.26%	21	24
Sorafenib	0.63 (0.48;0.83); P=0.0009	3.88%	57	57
Gefitinib	0.67 (0.55;0.82); P=0.0001	4.61%	24	24
(S)-Crizotinib	0.67 (0.59;0.77); P<0.0001	5.27%	30	30
Icotinib	0.68 (0.62;0.74); P<0.0001	5.56%	18	18
TGFBRII-Fc	0.69 (0.57;0.83); P<0.0001	4.78%	34	34
Fasudil hydrochloride	0.69 (0.66;0.73); P<0.0001	5.77%	133	135
Erlotinib	0.72 (0.56;0.91); P=0.006	4.20%	30	30
Imatinib	0.73 (0.67;0.80); P<0.0001	5.61%	55	61
Dasatinib	0.74 (0.58;0.93); P<0.0001	4.28%	260	266
Lapatinib	0.74 (0.61;0.90); P=0.003	4.62%	30	30
Sirolimus	0.79 (0.72;0.88); P<0.0001	5.49%	350	359
Tacrolimus (FK506)	0.79 (0.68;0.93); P=0.003	5.06%	24	24
BCG-3111	0.81 (0.78;0.83); P<0.0001	5.84%	18	18
Fasudil dichloroacetate	0.81 (0.74;0.86); P<0.0001	5.61%	39	31
IN-1233	0.81 (0.68;0.96); P=0.02	4.87%	41	41
Ruxolitinib	0.85 (0.77;0.94); P=0.001	5.51%	62	62
Nintedanib	0.85 (0.68;1.07); NS	4.38%	38	44
(R)-Crizotinib	1.33 (1.25;1.43); P<0.0001	5.70%	31	31

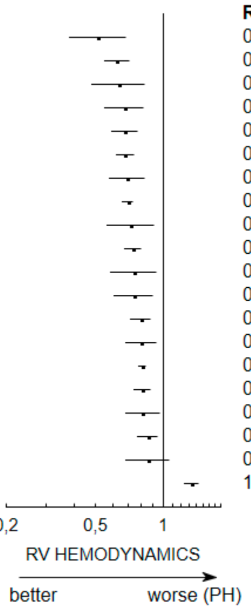

B

Drug	Risk ratio (95%CI)	Weight %	N1 (Treatment)	N2 (Placebo)
Sorafenib	0.65 (0.57;0.73); P<0.0001	4.73%	79	79
Seralutinib	0.68 (0.60;0.76); P<0.0001	5.01%	22	26
BCG-3111	0.70 (0.63;0.78); P<0.0001	5.39%	18	18
Fasudil dichloroacetate	0.70 (0.61;0.82); P<0.0001	3.77%	24	24
Gefitinib	0.71 (0.61;0.82); P<0.0001	3.90%	20	20
Fasudil hydrochloride	0.71 (0.66;0.76); P<0.0001	6.67%	300	312
Toceranib	0.76 (0.64;0.91); P=0.003	3.05%	24	24
(S)-Crizotinib	0.77 (0.50;1.18); NS	0.77%	18	17
BIBF1000	0.77 (0.65;0.90); P=0.001	3.46%	19	19
Imatinib	0.77 (0.73;0.82); P<0.0001	7.24%	229	243
TGFBRII-Fc	0.80 (0.76;0.83); P<0.0001	7.75%	24	24
Erlotinib	0.80 (0.66;0.96); P=0.02	2.87%	30	30
Sirolimus	0.80 (0.74;0.86); P<0.0001	6.33%	122	131
Masitinib	0.81 (0.74;0.89); P<0.0001	5.83%	24	24
Regorafenib	0.82 (0.77;0.89); P<0.0001	6.88%	13	13
Icotinib	0.82 (0.70;0.97); P=0.02	3.39%	15	15
Sunitinib	0.83 (0.76;0.91); P=0.0001	5.77%	25	27
IN-1233	0.84 (0.67;1.05); NS	2.25%	65	65
Lapatinib	0.85 (0.76;0.96); P=0.009	4.80%	30	30
Dasatinib	0.87 (0.67;1.15); NS	1.68%	35	40
Nintedanib	0.93 (0.75;1.16); NS	2.30%	24	24
Ruxolitinib	0.94 (0.87;1.02); NS	6.14%	24	24

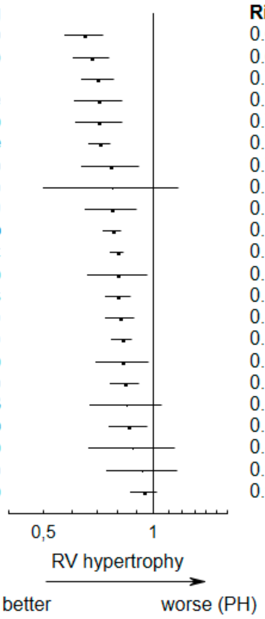

C

Drug	Risk ratio (95%CI)	Weight %	N1 (Treatment)	N2 (Placebo)
Fasudil hydrochloride	0.49 (0.42;0.57); P<0.0001	5.35%	256	259
Gefitinib	0.55 (0.44;0.69); P<0.0001	4.32%	76	76
Fasudil dichloroacetate	0.57 (0.48;0.68); P<0.0001	4.99%	24	24
TGFBRII-Fc	0.60 (0.50;0.73); P<0.0001	4.82%	36	36
Icotinib	0.66 (0.60;0.74); P<0.0001	5.94%	36	36
Masitinib	0.67 (0.57;0.78); P<0.0001	5.37%	24	24
Palbociclib	0.67 (0.53;0.86); P=0.001	4.16%	20	20
Imatinib	0.70 (0.63;0.77); P<0.0001	6.06%	206	209
Sorafenib	0.70 (0.63;0.78); P<0.0001	5.93%	93	93
Erlotinib	0.70 (0.56;0.88); P<0.0001	4.44%	60	60
Dasatinib	0.72 (0.45;1.16); NS	2.02%	38	43
PLX	0.74 (0.57;0.94); P=0.016	4.06%	32	32
Sirolimus	0.74 (0.67;0.83); P<0.0001	5.92%	192	98
K02288	0.75 (0.56;1.01); NS	3.51%	25	29
Lapatinib	0.79 (0.72;0.87); P<0.0001	6.12%	60	60
Sunitinib	0.84 (0.80;0.89); P<0.0001	6.44%	97	97
BCG-3111	0.88 (0.81;0.95); P=0.0006	6.28%	36	36
Ruxolitinib	0.93 (0.81;1.08); NS	5.44%	57	57
Toceranib	0.98 (0.89;1.07); NS	6.09%	48	48
(R)-Crizotinib	1.26 (0.87;1.83); NS	2.75%	24	24

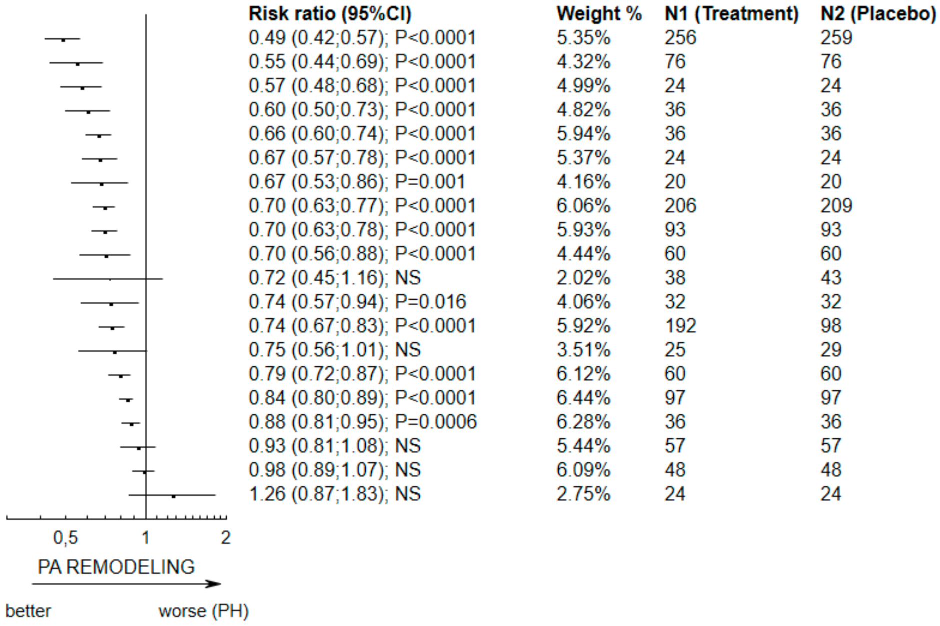

Supplementary Figure S2. Efficacy of selected small molecule protein kinase inhibitors in PH.

Tree plots demonstate the risk ratio (95%CI) for (A) – impairement in RV hemodynamics (combined end-point featured by right ventricle systolic pressure (RVSP) and mean pulmonary arterial pressure (mPAP)); (B) – RV hypertrophy; (C) – pulmonary artery (PA) remodeling. The risk ratio (R<1) indicates the reversal (improvement) in PH-related lesions.
